# Palmitoylation enhances the stability of porcine epidemic diarrhea virus spike protein by antagonizing its degradation via chaperone-mediated autophagy to facilitate viral proliferation

**DOI:** 10.1128/jvi.00347-25

**Published:** 2025-05-22

**Authors:** Qisheng Qian, Shuang-shuang Zhao, Lei Yang, Guangxu Xing, Yumei Chen, Chao Liang, Haili Wang, Rui Li, Songlin Qiao, Aiping Wang, Gaiping Zhang

**Affiliations:** 1 College of Veterinary Medicine, Jilin University 12510, Changchun, Jilin, China; 2 Institute for Animal Health, Henan Academy of Agricultural Sciences 74728, Zhengzhou, Henan, China; 3 Longhu Laboratory 693032 , Zhengzhou, China; 4 School of Life Sciences, Zhengzhou University 12636, Zhengzhou, Henan, China; St. Jude Children's Research Hospital, Memphis, Tennessee, USA

**Keywords:** porcine epidemic diarrhea virus, spike protein, palmitoylation, protein stability, chaperone-mediated autophagy

## Abstract

Porcine epidemic diarrhea (PED) is a highly pathogenic and infectious intestinal disease caused by the PED virus (PEDV) and has inflicted substantial economic losses on the global swine industry. Therefore, it is imperative to explore appropriate targets to restrain PEDV infection. PEDV spike (S) protein is crucial for viral infection and is regarded as an ideal target for the development of vaccines and antiviral therapeutics. Palmitoylation is a significant post-translational modification implicated in multiple viral replication cycles. Despite the fact that palmitoylation of certain coronavirus S proteins has been reported, the specific biological significance and underlying molecular mechanisms of PEDV S protein palmitoylation have not been fully defined. In the present study, we uncover that palmitoylation enhances the stability of PEDV S protein to promote viral proliferation. Mechanistically, we identify that a cysteine-rich region within the cytoplasmic tail of PEDV S protein is palmitoylated by the zinc finger Asp-His-His-Cys domain palmitoyltransferase 5 (ZDHHC5). We further illustrate that palmitoylation prevents the recognition of Lys-Phe-Glu-Arg-Gln (KFERQ)-like motif in PEDV S protein by heat shock cognate protein of 70 kDa (HSC70), thereby antagonizing its degradation via chaperone-mediated autophagy (CMA). Collectively, our findings underscore the importance of palmitoylation for PEDV pathogenesis and provide prospective targets for the development of antiviral interventions.

**IMPORTANCE:**

PEDV poses a serious threat to pig farming worldwide. As a consequence, a comprehensive investigation of PEDV pathogenesis is of great significance for the prevention and control of the virus. Here, we verify that ZDHHC5-mediated palmitoylation of PEDV S protein enhances its stability through impeding recognition by HSC70 and antagonizing degradation via CMA to facilitate viral propagation. Our findings highlight the important role of palmitoylation in PEDV proliferation and support palmitoylation as a promising target for the development of antiviral strategies.

## INTRODUCTION

Porcine epidemic diarrhea (PED), caused by PED virus (PEDV), is an acute and highly infectious intestinal disease and characterized by vomiting, severe diarrhea, anorexia, and dehydration (1). Swine of all ages are susceptible to PED, and especially, neonatal piglets suffer from the disease with a mortality rate up to 100% (2, 3). In 1971, PED was originally discovered in the United Kingdom and subsequently spread to several European countries (4). Since 2010, PED has become prevalent in China and broken out in other pig-raising countries, resulting in enormous economic losses (5–7). For example, the outbreak of PED in the United States in 2013 led to the deaths of seven million pigs, nearly 10% of the national swine population (8, 9).

PEDV is an enveloped single-stranded positive-sense RNA virus, belonging to the genus *Alphacoronavirus*, family *Coronaviridae*, order *Nidovirales* (10). Its genome has a length of approximately 28 kb and encodes non-structural proteins, an accessory protein, and structural proteins, including spike (S) protein, nucleocapsid (N) protein, membrane protein, and envelope protein (11, 12). Among them, the S protein plays a pivotal role in recognizing host cell receptors and mediating membrane fusion and is consequently considered a prospective antiviral target (13).

Palmitoylation is an important lipid post-translational modification (PTM) that involves the covalent attachment of medium-chain fatty acids (frequently palmitic acid and stearic acid) to specific cysteine residues of target proteins (14). Palmitoylation is typically catalyzed by members of the palmitoyltransferase family that contain the zinc-finger Asp-His-His-Cys domain (ZDHHC) (15) and plays critical roles in regulating protein subcellular localization, trafficking, stability, membrane association, and signal transduction (16, 17). Accumulating evidence has underscored the importance of palmitoylation for coronavirus (CoV) S proteins. For instance, palmitoylation has been shown to influence the intracellular trafficking, localization, and trimerization of severe acute respiratory syndrome coronavirus 2 (SARS-CoV-2) S protein (18–20). More importantly, palmitoylation of S proteins from transmissible gastroenteritis virus (TGEV), mouse hepatitis virus (MHV), severe acute respiratory syndrome coronavirus (SARS-CoV), and SARS-CoV-2 is essential for viral infectivity (20–24). However, the biological significance and the specific mechanisms of PEDV S protein palmitoylation have not been fully elaborated.

In this study, we initially demonstrated that PEDV S protein was palmitoylated via thioester bonds. We further identified ZDHHC5 as the primary host enzyme responsible for palmitoylation of the cysteine-rich region (CRR) in PEDV S protein cytoplasmic tail. Finally, we clarified that palmitoylation of PEDV S protein enhanced its stability by antagonism of degradation via chaperone-mediated autophagy (CMA) to promote viral proliferation.

## RESULTS

### PEDV S protein is palmitoylated through thioester bonds

**Fig 1:**
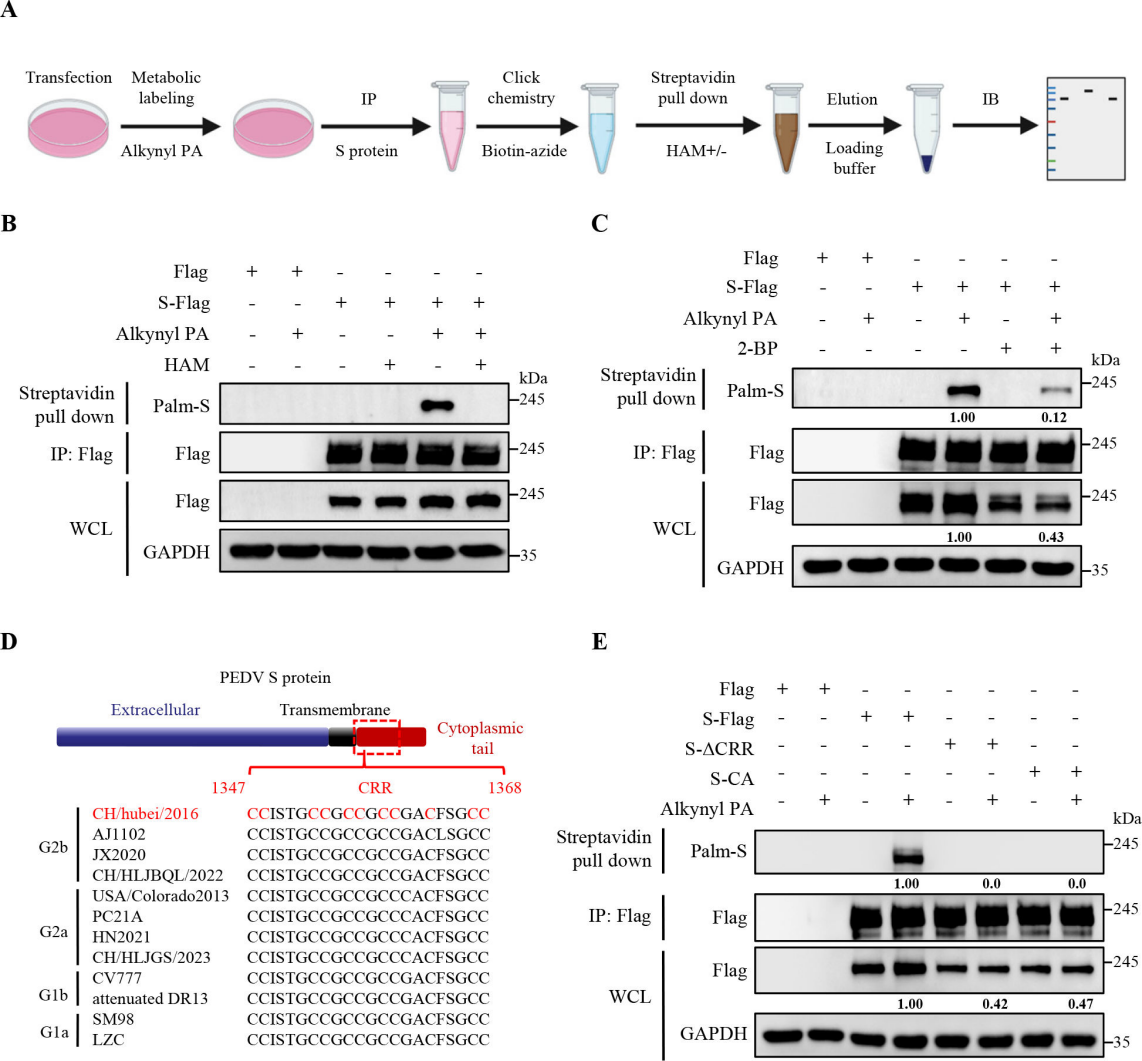
PEDV S protein is palmitoylated through thioester bonds. (**A**) A schematic for detecting protein palmitoylation by IP, click chemistry, and streptavidin pull-down assays. (**B**) HEK-293T cells were transfected with the plasmid encoding PEDV S-Flag or Flag-tagged empty vector for 24 h and metabolically labeled with alkynyl PA or DMSO as a control for 8 h. The supernatant of WCL was immunoprecipitated using anti-Flag magnetic beads. The beads were subjected to click chemistry, and treatment with or without HAM, followed by elution with glycine-HCl. The eluate was pulled down with streptavidin beads, and the precipitated proteins were analyzed by IB. (**C**) HEK-293T cells were transfected with the plasmid encoding PEDV S-Flag or Flag-tagged empty vector. The cells were treated with or without 40 µM 2-BP for 24 h and then metabolically labeled with Alkynyl PA or DMSO as a control for 8 h. Subsequent assays were performed as described for panel B. (**D**) Prediction of palmitoylation sites in PEDV S proteins. (**E**) HEK-293T cells were transfected with the plasmid encoding PEDV S-Flag, S-ΔCRR, or S-CA for 24 h. Subsequent assays were performed as described for panel B. The mean gray values of S protein were quantified using ImageJ software.

Initially, we performed cellular metabolic labeling using an alkyne-labeled palmitic acid analog (Alkynyl PA) in the PEDV S-Flag-overexpressed cells, followed by click chemistry with biotin azide and enrichment using streptavidin pulldown (Fig. 1A). Immunoblotting (IB) analysis showed that the S protein was labeled by Alkynyl PA, confirming its palmitoylation (Fig. 1B). Palmitoylation includes thioester bonds-linked S-palmitoylation, amide bonds-linked N-palmitoylation, and ester bonds-linked O-palmitoylation (25), and thioester bonds can be cleaved by hydroxylamine (HAM) treatment (26, 27). We conducted HAM treatment and found that the labeled alkynyl PA on the S protein was completely removed (Fig. 1B). These findings show that PEDV S protein is modified by S-palmitoylation. Conversely, we applied the non-cytotoxic 2-bromopalmitate (2-BP, Fig. S1A), a palmitoylation inhibitor (28, 29), and observed a reduction in the labeled Alkynyl PA (Fig. 1C), indicating that the palmitoylation of S protein was significantly inhibited. Subsequently, we utilized bioinformatic tools (CSS-Palm 4.0/GPS-Palm) to predict potential palmitoylation sites of PEDV S protein (30, 31). As shown in Fig. 1D, a total of 11 or 12 cysteine residues are predicted to be palmitoylated, which are all located in the so-called CRR in the cytoplasmic tail of S proteins from different PEDV genogroups. Consequently, we generated the mutant S protein with the deletion of the CRR (named as S-ΔCRR) or with substitution of these cysteine residues (C) by alanine ones (A; named as S-CA) and observed their complete loss of palmitoylation (Fig. 1E). Taken together, these results demonstrate that the CRR of PEDV S protein is modified by palmitoylation.

### Palmitoylation of PEDV S protein is mainly mediated by ZDHHC5

**Fig 2:**
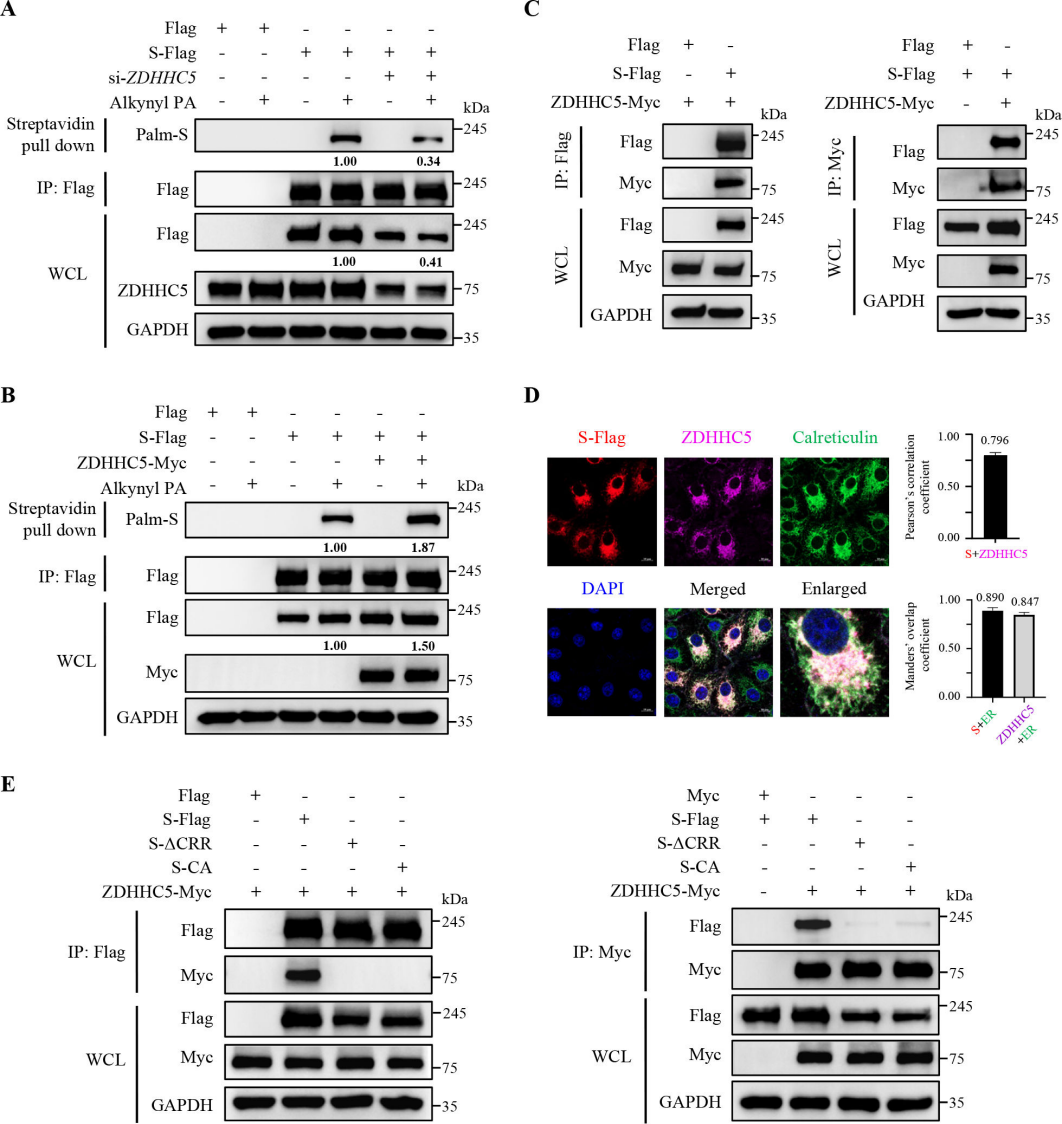
ZDHHC5 mainly mediates palmitoylation of PEDV S protein. (**A**) Vero cells were transfected with si-*ZDHHC5* or si-NC for 12 h and then were transfected with the plasmid encoding PEDV S-Flag or Flag-tagged empty vector for 24 h. Subsequent assays were performed as described for Fig. 1B. (**B**) HEK-293T cells were co-transfected with the plasmids encoding ZDHHC5-Myc and PEDV S-Flag or Flag-tagged empty vector for 24 h. Subsequent assays were performed as described for Fig. 1B. (**C**) HEK-293T cells were co-transfected with the plasmids encoding PEDV S-Flag and ZDHHC5-Myc, with Flag-tagged or Myc-tagged empty vector as control for 36 h. The supernatant of WCL was immunoprecipitated using anti-Flag magnetic beads or anti-Myc magnetic beads. Then, the precipitated proteins were analyzed by IB. (**D**) Vero cells were transfected with the plasmid encoding PEDV S-Flag for 24 h. The cells were visualized with the specific primary and secondary antibodies. Cell nuclei were stained with DAPI. Images were taken at a 630× magnification and representative of a single slice of a stack from three independent experiments. Representative images are shown. Scale bars, 10 µm. The co-localization was assessed by determination of the Pearson’s correlation coefficient and the Manders’ overlap coefficient using the JaCoP plugin in ImageJ software. The mean value ± SEM is representative of three individual pictures. (**E**) HEK-293T cells were co-transfected with the plasmids encoding PEDV S-Flag, S-ΔCRR, or S-CA, and ZDHHC5-Myc, with Flag-tagged or Myc-tagged empty vector as control for 36 h. The WCL was added with the protease inhibitor cocktail and was centrifuged to obtain the supernatant. The supernatant was immunoprecipitated using anti-Flag magnetic beads or anti-Myc magnetic beads. The precipitated proteins were analyzed by IB. The mean gray values of S protein were quantified using ImageJ software.

Palmitoylation is mediated by the ZDHHC family (15). To identify the specific ZDHHCs involved in the palmitoylation of PEDV S protein, we utilized a small interference RNA (siRNA) library targeting ZDHHC1 to ZDHHC24 (excluding ZDHHC11) to individually knock down each enzyme. We designed three pairs of siRNA duplexes targeting each ZDHHC and selected those with the highest knockdown efficiency for subsequent experiments (Fig. S2A). Subsequently, the indicated siRNAs or siRNA-negative control (si-NC) were transfected, followed by overexpression of PEDV S protein. IB results showed that *ZDHHC5* knockdown exhibited the most significant inhibition of the S protein palmitoylation (Fig. S2B). We further demonstrated that *ZDHHC5* knockdown decreased the palmitoylation level of PEDV S protein, whereas ZDHHC5 overexpression increased it (Fig. 2A and B). To validate their interaction, we co-expressed ZDHHC5-Myc and PEDV S-Flag and performed co-immunoprecipitation (co-IP). The results showed that ZDHHC5 interacted with S protein (Fig. 2C). Additionally, confocal microscopy analysis revealed the co-localization between ZDHHC5 and S protein at the endoplasmic reticulum ([ER], with calreticulin as a marker; Manders’ overlap coefficient >0.6, Fig. 2D), further supporting their interaction (Pearson’s correlation coefficient >0.5, Fig. 2D). Moreover, we found that both the S-ΔCRR and S-CA mutants lost their interaction with ZDHHC5 (Fig. 2E). These data indicate that the CRR of PEDV S protein is bound and palmitoylated by ZDHHC5.

### Palmitoylation of PEDV S protein enhances its stability

**Fig 3:**
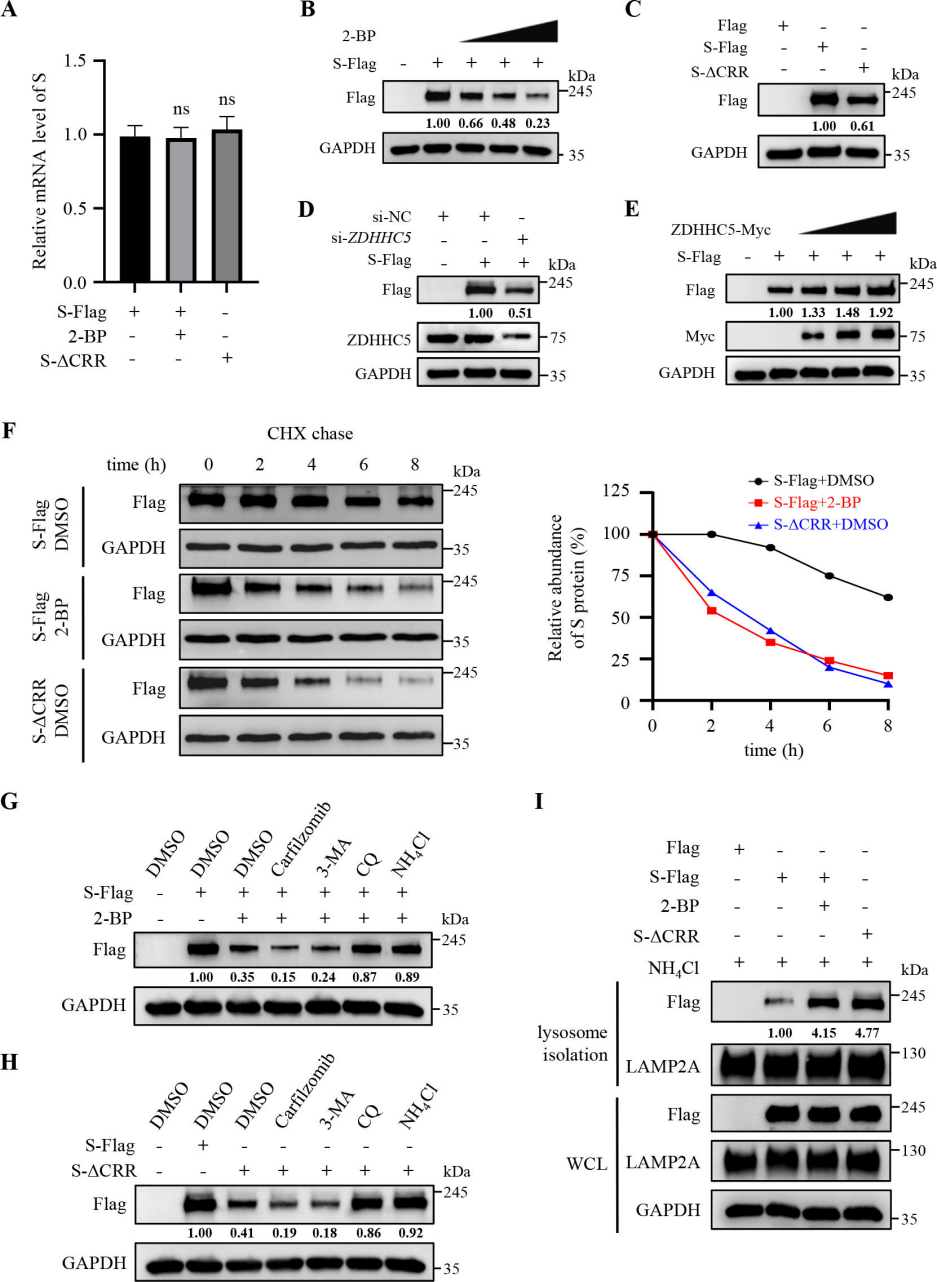
Palmitoylation of PEDV S protein enhances its stability. (**A**) Vero cells were transfected with the plasmid encoding PEDV S-Flag or S-ΔCRR. The cells were treated with or without 20 µM 2-BP for 36 h. The mRNA abundance of S protein was quantified by RT-qPCR. Statistical analysis was carried out using Student’s *t*-test. ns, not significant (*P* > 0.05). (**B**) Vero cells were transfected with the plasmid encoding PEDV S-Flag and were then treated with 2-BP (5 µM, 10 µM, and 20 µM) or DMSO for 36 h. The S protein levels were measured by IB. (**C**) Vero cells were transfected with the plasmid encoding PEDV S-Flag, S-ΔCRR, or Flag-tagged empty vector for 36 h. The S protein levels were measured by IB. (**D**) Vero cells were transfected with si-*ZDHHC5* or si-NC for 12 h and were then transfected with the plasmid encoding PEDV S-Flag or Flag-tagged empty vector for 24 h. The S protein levels were measured by IB. (**E**) Vero cells were co-transfected with the plasmids encoding S-Flag and ZDHHC5-Myc (0.5 µg, 1 µg, and 2 µg) for 36 h. The S protein levels were measured by IB. (**F**) Vero cells were transfected with the plasmid encoding PEDV S-Flag or S-ΔCRR. The S-Flag-overexpressed cells were treated with or without 20 µM 2-BP for 24 h. Then, the cells were stimulated with CHX (1 µg/mL) and lysed at the indicated time points (0 h, 2 h, 4 h, 6 h, and 8 h). The samples were analyzed by IB. (**G**) Vero cells were transfected with the plasmid encoding PEDV S-Flag or Flag-tagged empty vector. The cells were treated with DMSO or 20 µM 2-BP, and then co-incubated with DMSO, 3-MA (2 mM), carfilzomib (20 nM), NH_4_Cl (0.5 mM), or CQ (40 µM) for 36 h. The cells were lysed and analyzed by IB. (**H**) Vero cells were transfected with the plasmid encoding PEDV S-Flag, S-ΔCRR, or Flag-tagged empty vector. The cells were treated with DMSO, 3-MA (2 mM), carfilzomib (20 nM), NH_4_Cl (0.5 mM), or CQ (40 µM) for 36 h. The cells were lysed and analyzed by IB. (**I**) Vero cells were transfected with the plasmid encoding PEDV S-Flag, S-ΔCRR, or Flag-tagged empty vector. At 6 h post-transfection, the S-Flag-overexpressed groups were treated with or without 20 µM 2-BP, and all the cells were treated with NH_4_Cl (0.5 mM) for 24 h. The lysosomes were isolated from these cells and analyzed by IB. The mean gray values of S protein were quantified using ImageJ software.

We next explored the biological significance of palmitoylation on PEDV S protein. As illustrated in Fig. 1C, E and 2A, the palmitoylation deficiency reduced the protein levels of PEDV S protein in whole-cell lysates (WCLs). Therefore, we hypothesized that palmitoylation probably participates in maintaining the stability of PEDV S protein. To prove our hypothesis, we determined that treatment with 2-BP or deletion of the CRR didn’t affect the mRNA abundance of S protein but led to a reduction in their protein levels (Fig. 3A through C). Furthermore, *ZDHHC5* knockdown resulted in a distinct drop in the S protein level (Fig. 3D), whereas ZDHHC5 overexpression increased its protein levels in a dose-dependent manner (Fig. 3E). These results show that palmitoylation is critical for maintaining the stability of PEDV S protein against degradation. To monitor the degradation dynamics of PEDV S protein, we conducted a cycloheximide (CHX) chase assay according to recent studies (32, 33). The decay analyses indicated that the apparent half-life of S protein was approximately 8 h, whereas treatment with 2-BP or deletion of the CRR remarkably shortened its half-life to approximately 2 h (Fig. 3F). These results substantiate that palmitoylation of PEDV S protein strengthens its stability.

As the autophagy-lysosome pathway and ubiquitin-proteasome system are two major host cellular protein degradation routes in eukaryotic cells (34, 35), we examined whether these two routes took effect on the palmitoylation deficiency-induced degradation of PEDV S protein. As shown in Fig. 3G and H, neither the proteasome inhibitor carfilzomib nor the autophagy inhibitor 3-methyladenine (3-MA) impeded the 2-BP- or S-ΔCRR-induced S protein degradation. In contrast, two lysosomal inhibitors, ammonium chloride (NH_4_Cl) and chloroquine (CQ), were able to restore the S protein levels (Fig. 3G and H). Furthermore, we overexpressed S-Flag with or without 2-BP treatment, or S-ΔCRR, and isolated lysosomes in these cells. IB results showed that treatment with 2-BP or deletion of the CRR directed more S protein into lysosomes (Fig. 3I). These data verify that palmitoylation of PEDV S protein prevents its degradation via the lysosomal pathway.

### The KFERQ-like motif (QVDRL) in PEDV S protein is recognized and bound by HSC70

**Fig 4:**
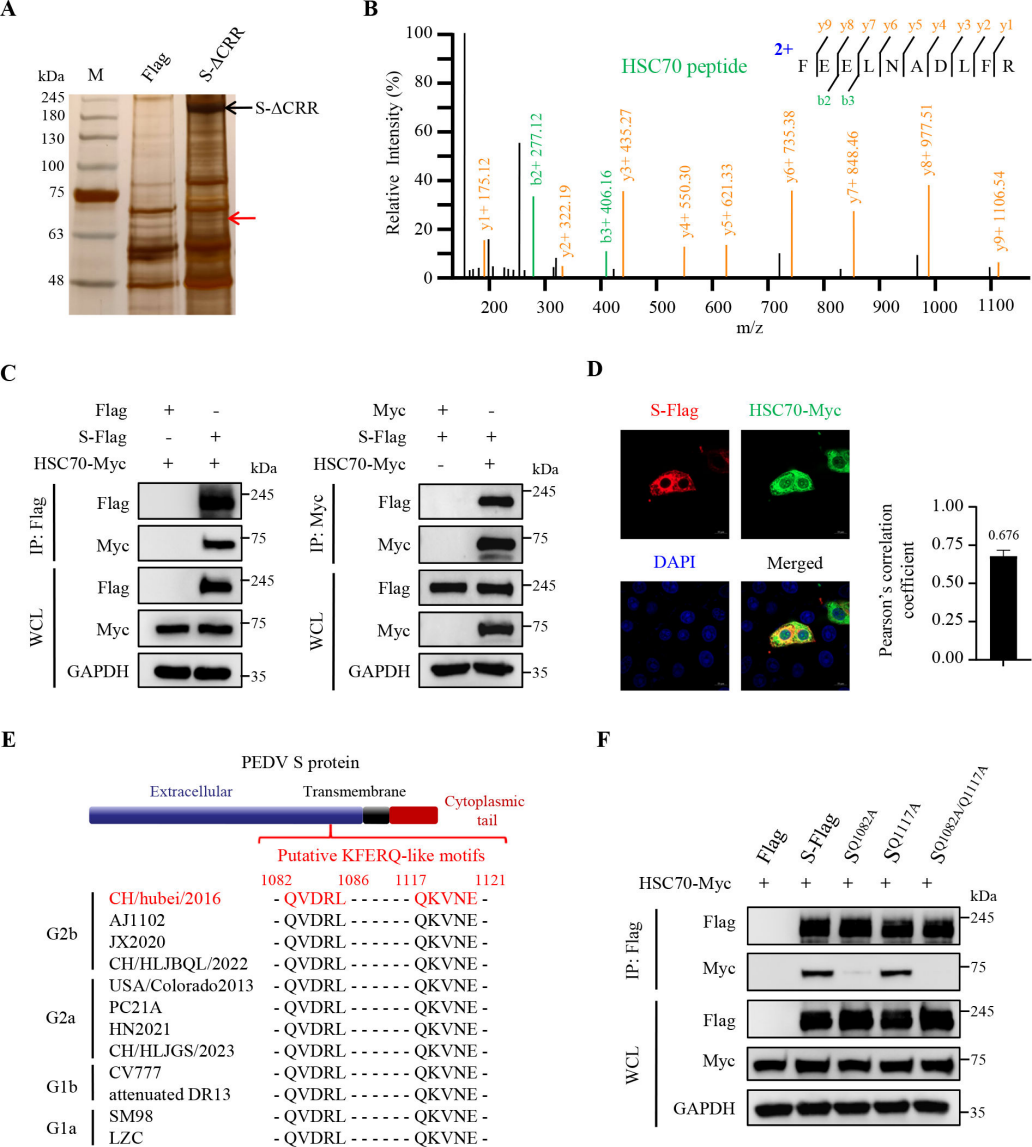
The KFERQ-like motif (QVDRL) in PEDV S protein is recognized and bound by HSC70. (**A**) Vero cells were transfected with the plasmid encoding PEDV S-ΔCRR or Flag-tagged empty vector for 36 h. The proteins were immunoprecipitated in WCL using anti-Flag magnetic beads, and then the associated proteins were separated by 7.5% SDS-PAGE and stained with silver. The black arrow indicated the expressed S-ΔCRR, and the red arrow indicated a different immunoprecipitated protein band in the S-ΔCRR-expressed cells. Lane M, protein marker. (**B**) The silver-stained protein band indicated by the red arrow was subjected to LC-MS/MS. (**C**) HEK-293T cells were co-transfected with the plasmids encoding PEDV S-Flag and HSC70-Myc, with Flag-tagged or Myc-tagged empty vector as control for 36 h. The supernatant of WCL was immunoprecipitated using anti-Flag magnetic beads or anti-Myc magnetic beads. The precipitated proteins were analyzed by IB. (**D**) Vero cells were co-transfected with the plasmids encoding PEDV S-Flag and HSC70-Myc for 24 h. The cells were visualized with the specific primary and secondary antibodies. Cell nuclei were stained with DAPI. Images were taken at a 630× magnification and representative of a single slice of a stack from three independent experiments. Representative images are shown. Scale bars, 10 µm. The co-localization was assessed by determination of the Pearson’s correlation coefficient using the JaCoP plugin in ImageJ software. The mean value ± SEM is representative of three individual enlarged pictures. (**E**) Analyses of KFERQ-like motifs in PEDV S proteins. The KFERQ-like motif typically consists of constant glutamine (Q) flanked on one side of the pentapeptide sequence, followed by one or two positively charged amino residues (lysine, [K] or arginine, [R]), one or two hydrophobic amino residues (phenylalanine, [F]; isoleucine, [I]; leucine, [L]; or valine, [V]), and one negatively charged amino residue (aspartate, [D] and glutamic acid, [E]) (36). (**F**) HEK-293T cells were co-transfected with the plasmids encoding HSC70-Myc and PEDV S-Flag, S^Q1082A^, S^Q1117A^, S^Q1082A/Q1117A^, or Flag-tagged empty vector. At 36 h post-transfection, the supernatant of WCL was immunoprecipitated using anti-Flag magnetic beads. The precipitated proteins were analyzed by IB.

To further dissect the underlying mechanism by which palmitoylation protected PEDV S protein from degradation, we conducted IP and silver staining in the S-ΔCRR-overexpressed cells. A distinct immunoprecipitated protein band marked by the red arrow was observed in the S-ΔCRR-overexpressed cells (Fig. 4A), and the predominant S-ΔCRR-associated protein was identified as heat shock cognate protein of 70 kDa (HSC70) by liquid chromatography and tandem mass spectrometry (LC-MS/MS; Fig. 4B). We further confirmed the interaction between recombinant PEDV S protein and HSC70 by co-IP and confocal microscopy, respectively (Fig. 4C and D). HSC70 is well established to recognize the Lys-Phe-Glu-Arg-Gln (KFERQ)-like motifs of target proteins and participate in the lysosomal degradation of these proteins, namely CMA (36–38). We analyzed the amino acid sequences of PEDV S protein from different genogroups and found two conserved putative KFERQ-like motifs, suggesting it as a CMA substrate (Fig. 4E). To determine whether PEDV S protein was recognized by HSC70 dependent on the KFERQ-like motifs, we substituted these glutamine residues (Q) with alanine ones (A) to generate three mutants (named as S^Q1082A^, S^Q1117A^, and S^Q1082A/Q1117A^). We observed that the mutants S^Q1082A^ and S^Q1082A/Q1117A^ abolished the interaction between S protein and HSC70 (Fig. 4F). The results indicate that HSC70 interacts with PEDV S protein by recognizing its KFERQ-like motif (QVDRL).

### Palmitoylation of PEDV S protein prevents its degradation via CMA

**Fig 5:**
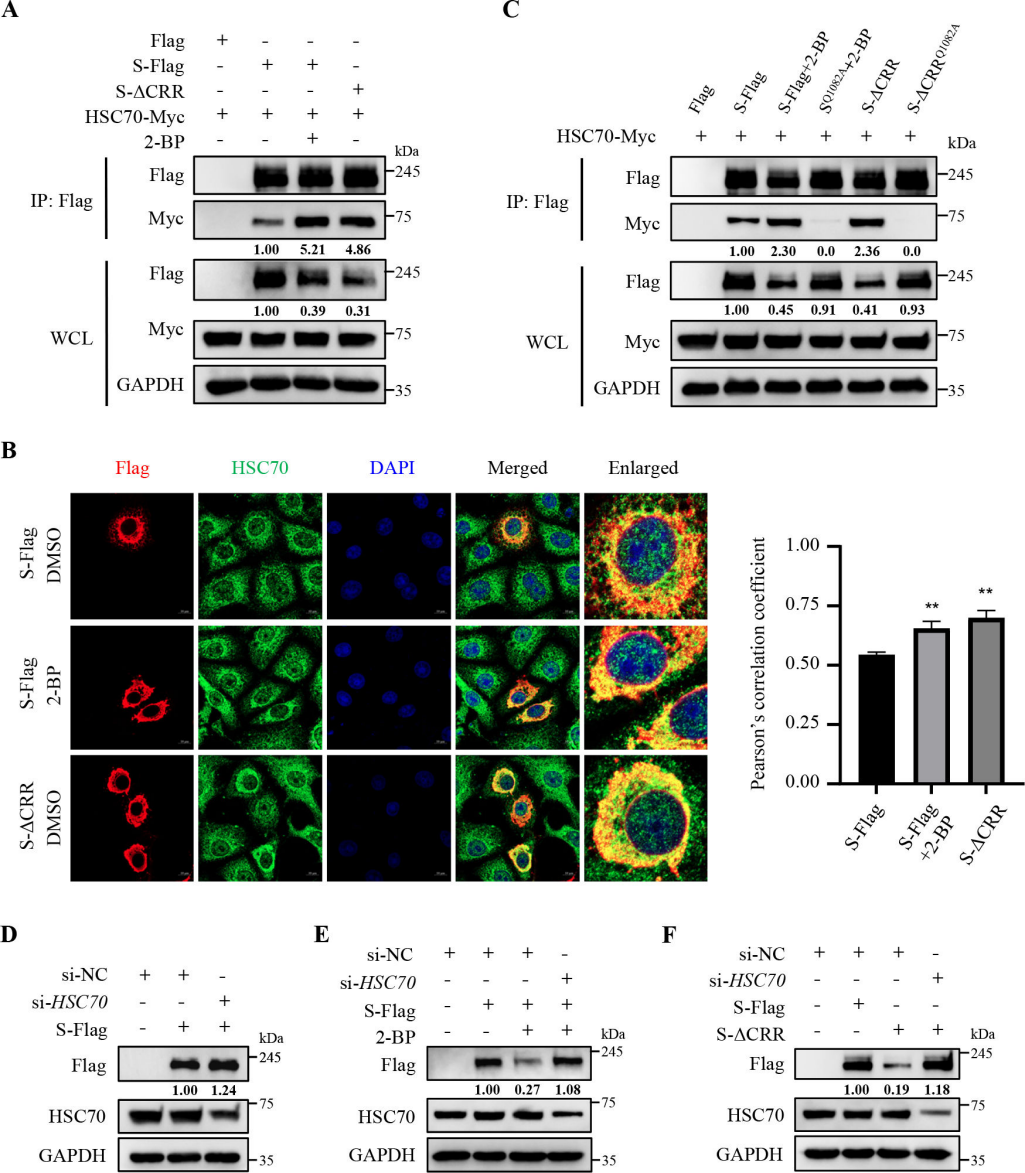
Palmitoylation of PEDV S protein prevents its QVDRL motif from being recognized by HSC70. (**A**) HEK-293T cells were transfected with the plasmids encoding HSC70-Myc and PEDV S-Flag, S-ΔCRR, or Flag-tagged empty vector. At 6 h post-transfection, the S-Flag-overexpressed cells were treated with or without 40 µM 2-BP. After 36 h post-transfection, the supernatant of WCL was immunoprecipitated using anti-Flag magnetic beads, and the precipitated proteins were analyzed by IB. (**B**) Vero cells were transfected with the plasmid encoding PEDV S-Flag or S-ΔCRR. At 6 h post-transfection, the S-Flag-overexpressed groups were treated with or without 20 µM 2-BP. At 24 h post-transfection, S-Flag, S-ΔCRR, or HSC70 was visualized with the specific primary and secondary antibodies. Cell nuclei were stained with DAPI. Images were taken at a 630× magnification and representative of a single slice of a stack from three independent experiments. Representative images are shown. Scale bars, 10 µm. The co-localization was assessed by determination of the Pearson’s correlation coefficient using the JaCoP plugin in ImageJ software. The mean value ± SEM is representative of three individual enlarged pictures. Statistical analysis was carried out using Student’s *t*-test. **P < 0.01. (**C**) HEK-293T cells were co-transfected with the plasmids encoding HSC70-Myc and PEDV S-Flag, S-ΔCRR, S^Q1082A^, S-ΔCRR^Q1082A^, or Flag-tagged empty vector. At 6 h post-transfection, the S-Flag-overexpressed and the S^Q1082A^-overexpressed cells were treated with or without 40 µM 2-BP. Subsequent assays were performed as described for panel A. (D through F) Vero cells were transfected with si-*HSC70* or si-NC. At 12 h post-transfection, the cells were transfected with the plasmid encoding PEDV S-Flag, S-ΔCRR, or Flag-tagged empty vector, and treated with or without 20 µM 2-BP for another 24 h. The cells were lysed and analyzed by IB. The mean gray values of S protein and HSC70-Myc were quantified using ImageJ software.

**Fig 6:**
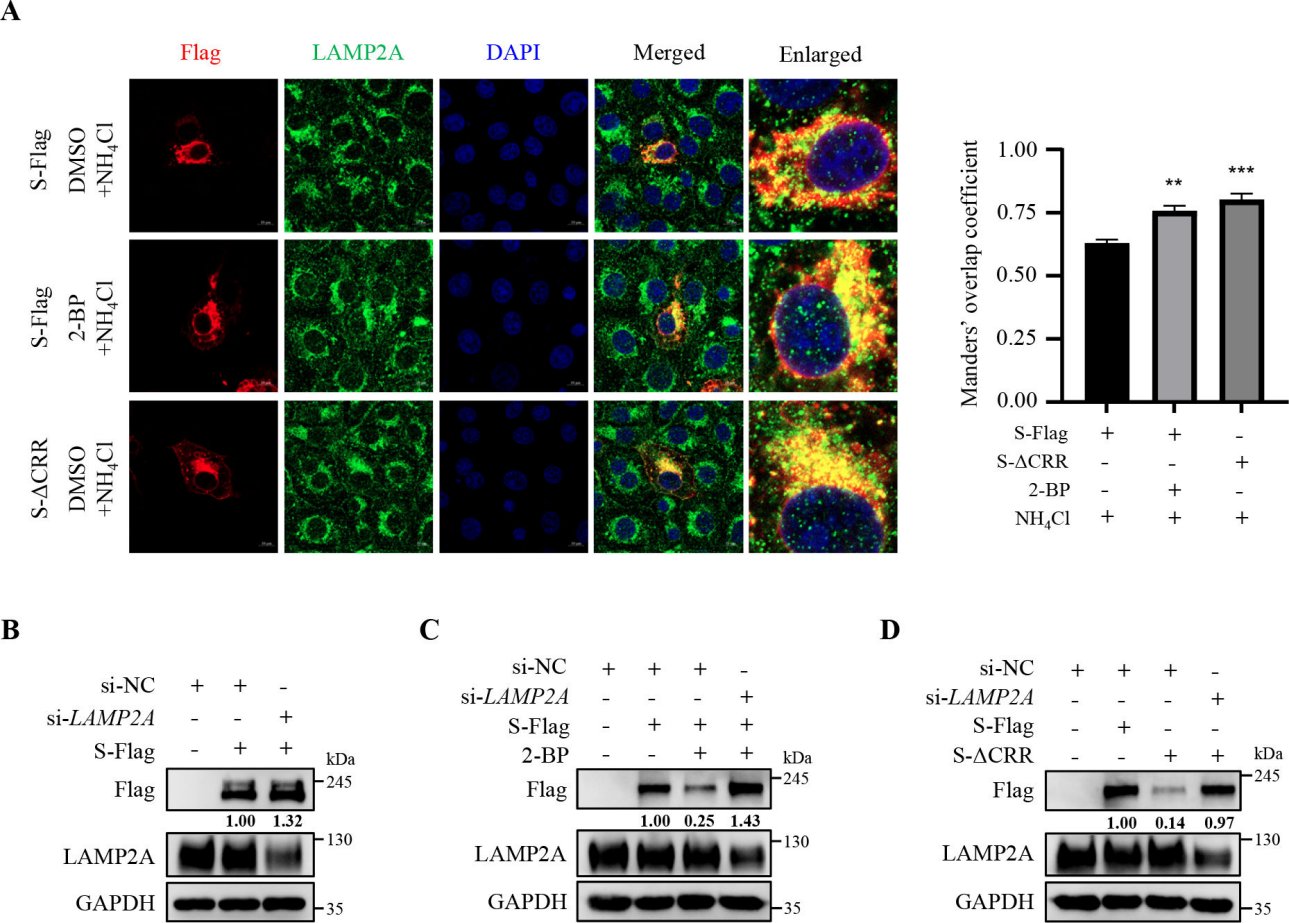
Palmitoylation of PEDV S protein prevents its degradation via CMA. (**A**) Vero cells were transfected with the plasmid encoding PEDV S-Flag or S-ΔCRR. At 6 h post-transfection, the S-Flag-overexpressed cells were treated with or without 20 µM 2-BP and then treated with NH_4_Cl (0.5 mM). At 24 h post-transfection, S-Flag, S-ΔCRR, or LAMP2A was visualized with the specific primary and secondary antibodies. Cell nuclei were stained with DAPI. Images were taken at a 630× magnification and representative of a single slice of a stack from three independent experiments. Representative images are shown. Scale bars, 10 µm. The co-localization was assessed by determination of the Manders’ overlap coefficient using the JaCoP plugin in ImageJ software. The mean value ± SEM is representative of three individual enlarged pictures. Statistical analysis was carried out using Student’s *t*-test. **P < 0.01; ***P < 0.001. (B through D) Vero cells were transfected with si-*LAMP2A* or si-NC. At 12 h post-transfection, the cells were transfected with the plasmid encoding PEDV S-Flag, S-ΔCRR, or Flag-tagged empty vector and treated with or without 20 µM 2-BP for another 24 h. The cells were lysed and analyzed by IB. The mean gray values of S protein were quantified using ImageJ software.

Based on the above results, we continued to investigate whether palmitoylation affected the degradation of PEDV S protein by influencing its recognition by HSC70. As depicted in Fig. 5A, treatment with 2-BP or deletion of the CRR significantly strengthened the interaction between HSC70 and S protein and correspondingly reduced its protein levels. Confocal microscopy further validated that treatment with 2-BP or deletion of the CRR enhanced their interaction (Fig. 5B). However, mutation of the QVDRL motif reversed the palmitoylation deficiency-induced degradation of S protein by disrupting its interaction with HSC70 (Fig. 5C). Moreover, we proved that *HSC70* knockdown raised the levels of S protein (Fig. 5D) and effectively reversed the 2-BP- or S-ΔCRR-induced degradation of S protein (Fig. 5E and F). These results demonstrate that palmitoylation of PEDV S protein prevents its recognition by HSC70 to antagonize degradation. During CMA-mediated protein degradation, the substrate-HSC70 complex is mediated by lysosome-associated membrane protein 2A (LAMP2A) into lysosomes for degradation (37, 38). Confocal microscopy revealed an increased co-localization of S protein with LAMP2A upon treatment with 2-BP or deletion of the CRR, indicating its enhanced lysosomal localization (Fig. 6A). In contrast, *LAMP2A* knockdown reversed the degradation of palmitoylation-deficient S protein (Fig. 6B through D), supporting the involvement of LAMP2A in S protein degradation. Collectively, all these results corroborate that palmitoylation of PEDV S protein strengthens its stability by antagonizing its degradation via CMA.

### Palmitoylation of PEDV S protein prevents its degradation via CMA during infection

**Fig 7:**
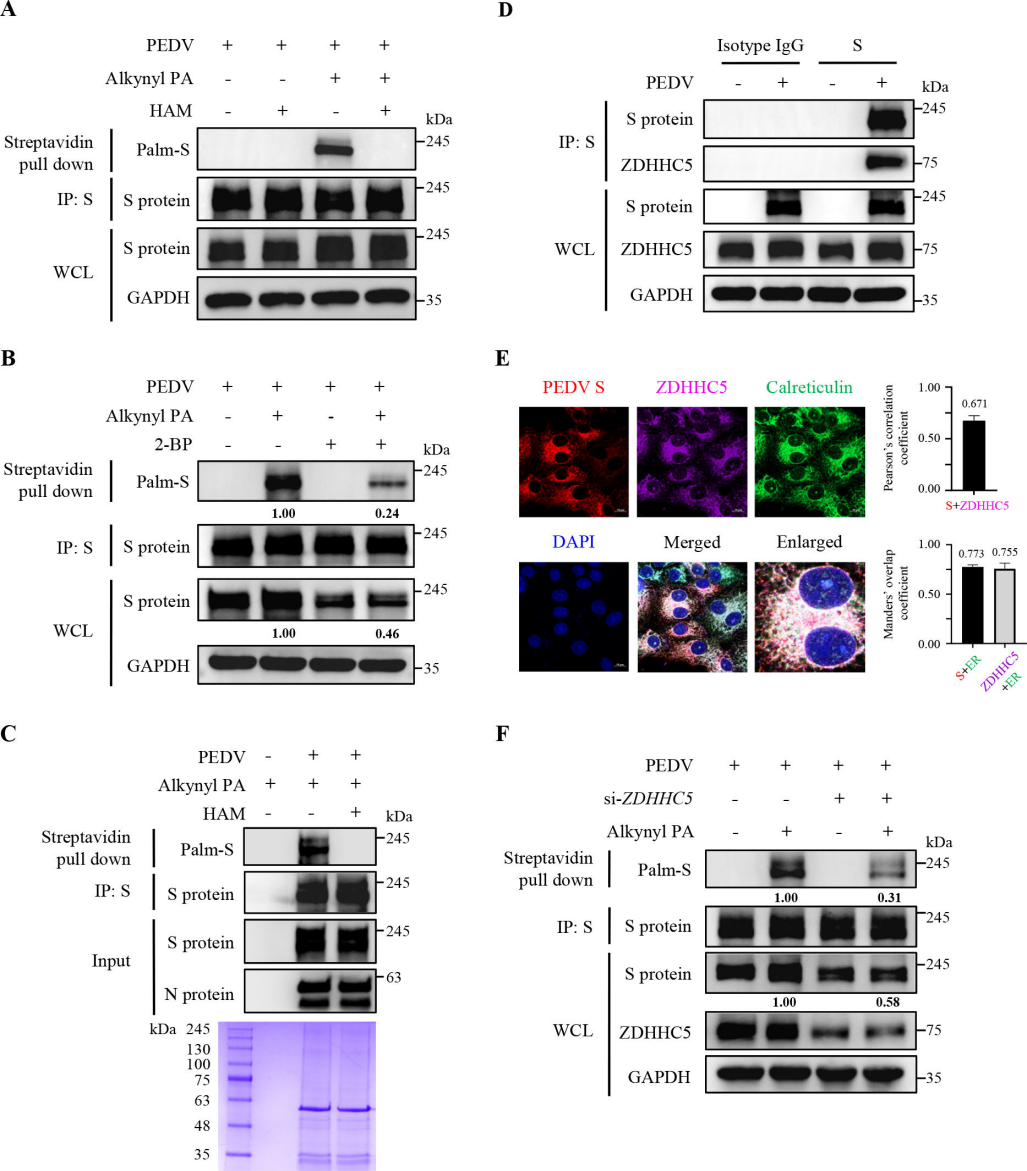
Palmitoylation of PEDV S protein was mediated by ZDHHC5 during infection. (**A**) Vero cells were infected with PEDV at a multiplicity of infection (MOI) of 0.05 for 12 h and metabolically labeled with alkynyl PA or DMSO as a control for 8 h. The supernatant of WCL was immunoprecipitated with 10 µg anti-PEDV S mAb and 100 µL protein G magnetic beads. The beads were subjected to click chemistry, and treatment with or without HAM, followed by elution with glycine-HCl. The eluate was pulled down with streptavidin beads, and the precipitated proteins were analyzed by IB. (**B**) Vero cells were infected with PEDV at 0.05 MOI for 12 h. The cells were treated with or without 6 µM 2-BP and metabolically labeled with Alkynyl PA or DMSO as a control for 8 h. Subsequent assays were performed as described for panel A. (**C**) PEDV was propagated in Vero cells at 0.05 MOI for 12 h and metabolically labeled with 50 µM alkynyl PA for 8 h. The mature virions were purified from the culture supernatant by sucrose gradient supercentrifugation and detected by SDS-PAGE. Subsequent assays were performed as described for panel A. (**D**) Vero cells were infected with PEDV at 0.25 MOI for 8 h and then lysed. Using S protein as bait, the precipitated proteins were analyzed by IB. Isotype IgG antibody was used as a negative control. (**E**) Vero cells were infected with PEDV at 0.25 MOI for 8 h. Then, PEDV S protein, ZDHHC5, and calreticulin were visualized with the specific primary and secondary antibodies. Subsequent assays were performed as described for Fig. 2D. (**F**) Vero cells were transfected with si-*ZDHHC5* or si-NC for 24 h and were then infected with PEDV at 0.05 MOI for 12 h. Subsequent assays were performed as described for panel A. The mean gray values of S protein were quantified using ImageJ software.

**Fig 8:**
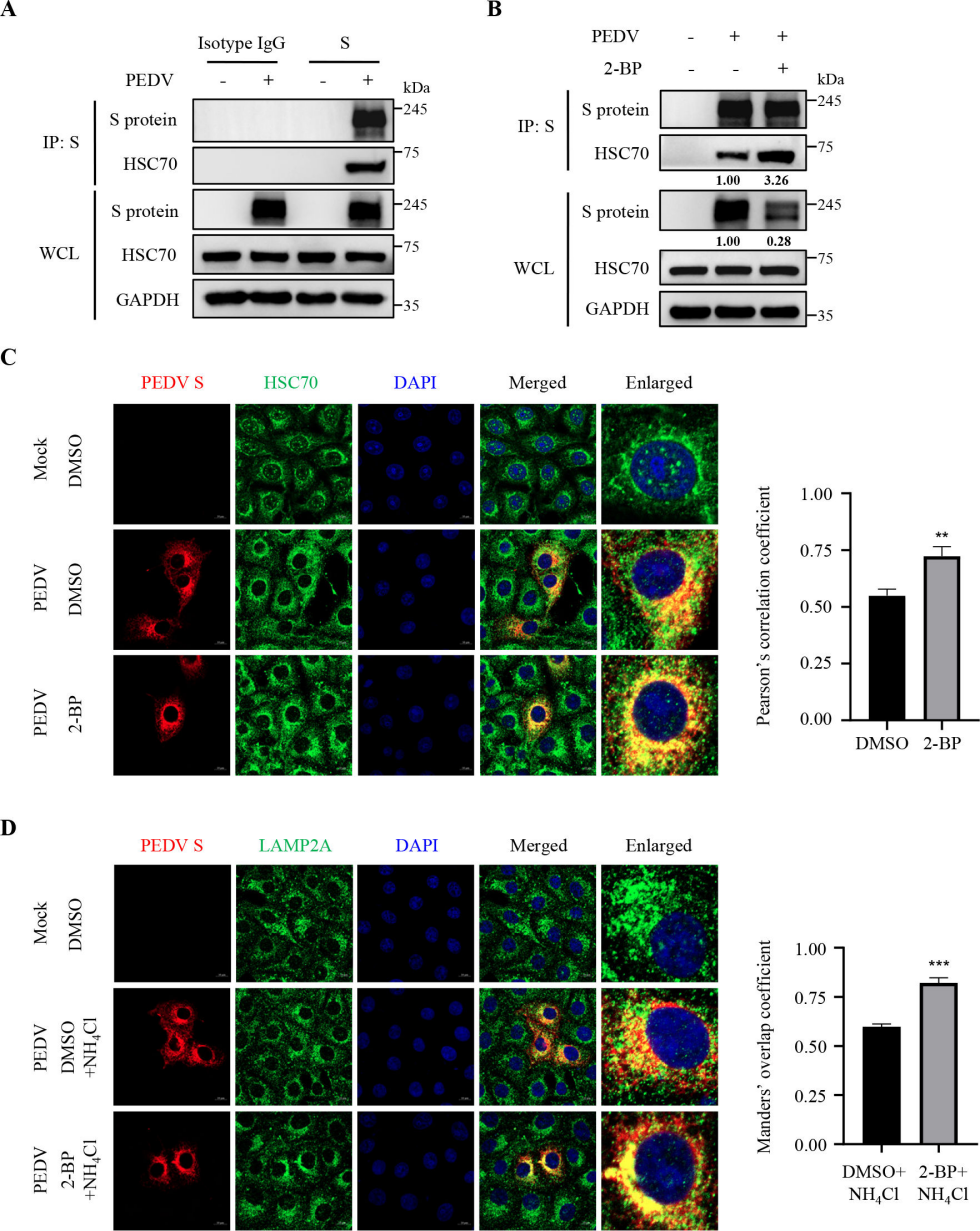
Palmitoylation of PEDV S protein prevents its degradation via CMA during infection. (**A**) Vero cells were infected with PEDV at 0.25 MOI for 8 h and then lysed. The supernatant of WCL was immunoprecipitated with anti-S mAb, and the precipitated samples were analyzed by IB. Isotype IgG antibody was used as a negative control. (**B**) Vero cells were mock-infected or infected by PEDV at 0.25 MOI, treated with or without 6 µM 2-BP for 8 h and then lysed. The supernatant of WCL was immunoprecipitated with anti-S mAb, and the eluted samples were measured by IB. (**C**) Vero cells were mock-infected or infected with PEDV at 0.25 MOI and treated with or without 6 µM 2-BP for 8 h. Then, PEDV S protein and HSC70 were visualized with the specific primary and secondary antibodies. Subsequent assays were performed as described for Fig. 5B. Statistical analysis was carried out using Student’s *t*-test. **P < 0.01. (**D**) Vero cells were mock-infected or infected with PEDV at 0.25 MOI and then treated with or without 6 µM 2-BP and NH_4_Cl (0.5 mM) for 8 h. Then, PEDV S protein and LAMP2A were visualized with the specific primary and secondary antibodies. Subsequent assays were performed as described for Fig. 6A. Statistical analysis was carried out using Student’s *t*-test. ***P < 0.001. The mean gray values of S protein and HSC70 were quantified using ImageJ software.

Next, we validated the biological significance and mechanisms of palmitoylation of PEDV S protein during infection. We first confirmed that the S protein underwent S-palmitoylation via thioester bonds in the PEDV-infected African green monkey kidney epithelial Vero cells (Fig. 7A), whereas non-cytotoxic 2-BP treatment observably decreased the palmitoylation and protein levels of S protein during infection (Fig. 7B; Fig. S1B). Additionally, we proved that the S protein incorporated into mature PEDV virions was modified by S-palmitoylation (Fig. 7C). Moreover, we corroborated the interaction between endogenous ZDHHC5 and S protein in the PEDV-infected cells using IP and confocal microscopy (Fig. 7D and E), and *ZDHHC5* knockdown lowered the palmitoylation and protein levels of S protein (Fig. 7F). As shown in Fig. 8A and B, we substantiated that PEDV S protein interacted with HSC70, and 2-BP treatment significantly enhanced their interaction, leading to the degradation of S protein. Confocal microscopy also validated that 2-BP treatment increased their interaction (Fig. 8C) and promoted the co-localization of S protein with LAMP2A (Fig. 8D). Considering swine serve as the natural host for PEDV, we conducted these assays in IPEC-J2 cells, derived from porcine intestinal epithelial cells, and observed the similar results (Fig. S1G and S3). Taken together, these data underscore that palmitoylation of PEDV S protein enhances its protein stability by antagonizing the CMA-mediated degradation during infection.

### Palmitoylation of PEDV S protein is critical for viral proliferation

**Fig 9:**
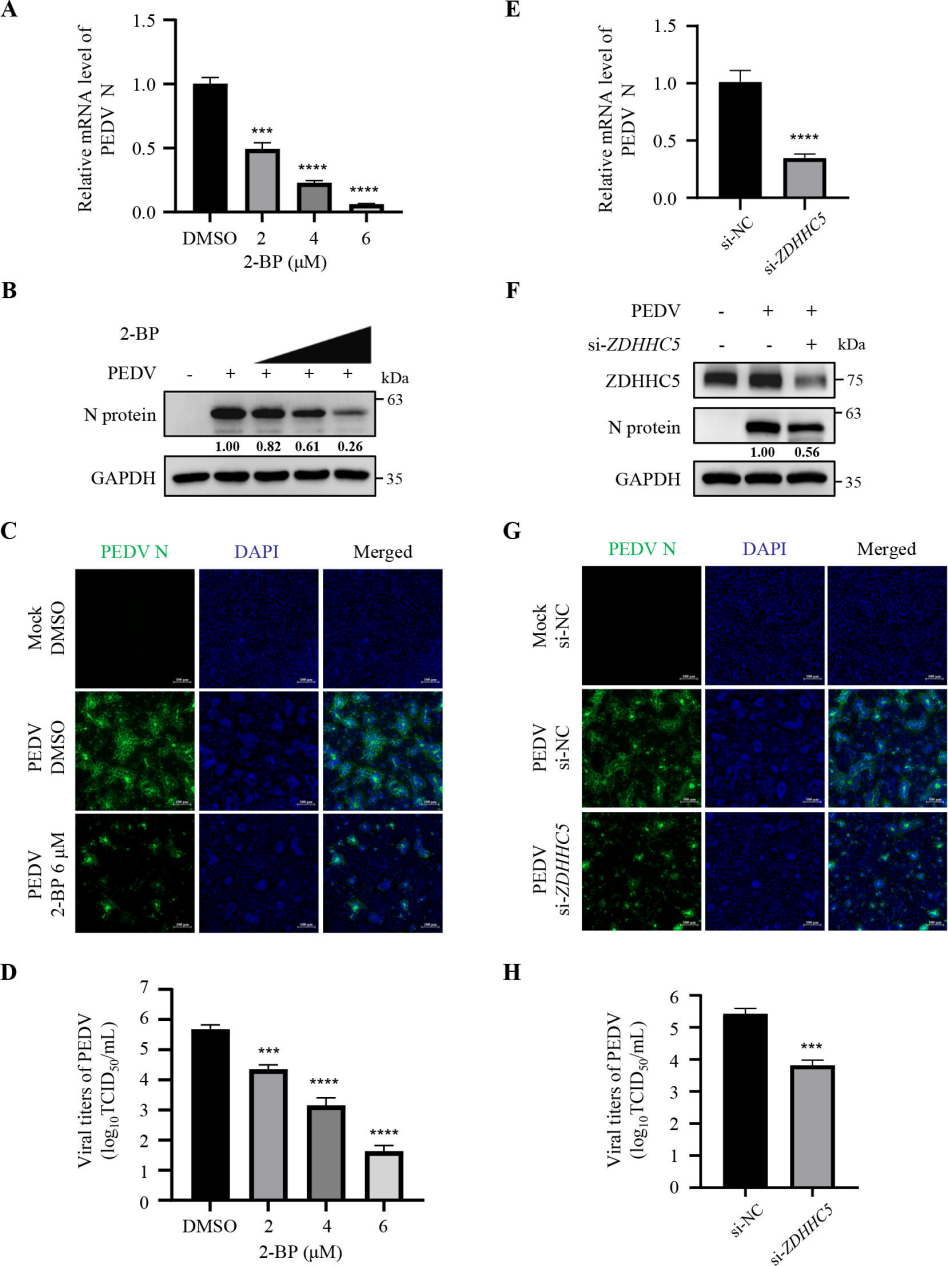
Palmitoylation of PEDV S protein is critical for viral proliferation. (A through D) Vero cells were mock-infected or infected with PEDV at 0.05 MOI and co-incubated with 2-BP (2 µM, 4 µM, or 6 µM) or DMSO for 24 h. (E–H) Vero cells were transfected with si-*ZDHHC5* or si-NC for 24 h and inoculated with PEDV at 0.05 MOI for 24 h. (A and E) The viral RNA abundance was detected using RT-qPCR. Statistical analysis was carried out using Student’s *t*-test. ***P < 0.001; ****P < 0.0001. (B and F) The PEDV N protein levels were analyzed by IB. (C and G) PEDV infectivity was detected by IFA. IFA images were taken by confocal microscopy, and the numbers of PEDV N-expressed and DAPI-stained cells were counted using ImageJ software. The ratio of PEDV-infected cells to total was calculated. Scale bars, 100 µm. (D and H) PEDV titers were measured by assessing TCID_50_. Statistical analysis was carried out using Student’s *t*-test. ***P < 0.001; ****P < 0.0001. The mean gray values of N protein were quantified using ImageJ software.

To investigate the impact of S protein palmitoylation on PEDV proliferation, we inoculated PEDV into Vero cells and treated them with non-cytotoxic 2-BP, followed by real-time quantitative PCR (RT-qPCR) to evaluate PEDV proliferation. The RT-qPCR results revealed a dose-dependent reduction in PEDV RNA abundance upon 2-BP treatment (Fig. 9A). IB analyses further confirmed a decrease in PEDV N protein levels following 2-BP treatment (Fig. 9B). In addition, 2-BP treatment led to a substantial decline in PEDV infectivities (~75% as detected by indirect immunofluorescence assay [IFA], Fig. 9C). Furthermore, the titers of progeny PEDV were diminished by 2-BP treatment, as evidenced by reduced TCID_50_ (50% tissue culture infected dose). Specifically, treatment with 6 µM 2-BP led to a more than 1000-fold reduction in progeny PEDV titers (>3 log_10_TCID_50_/mL; Fig. 9D). To further verify the effect of PEDV S protein palmitoylation on viral propagation, we took efforts to construct a recombinant PEDV with deletion of the CRR in its S protein but failed to rescue it (data not shown). Therefore, we proceeded to knock down ZDHHC5 in Vero cells and found that *ZDHHC5* knockdown inhibited PEDV proliferation (Fig. 9E through H). In order to exclude the possibility of si-*ZDHHC5* cytotoxicity, we performed the cell viability assay in Vero cells. The results showed that the cell viability of si-*ZDHHC5*-transfected cells was comparable to that of si-NC-transfected ones (Fig. S1H), suggesting that *ZDHHC5* knockdown inhibited viral multiplication by reducing the palmitoylation of PEDV S protein rather than due to its cytotoxicity. Consistently, PEDV propagation was markedly suppressed by 2-BP treatment and *ZDHHC5* knockdown in IPEC-J2 cells (Fig. S1I and S4). PEDV strains are mainly divided into four genogroups: G1a, G1b, G2a, and G2b (39). As the PEDV strain CH/hubei/2016 used in the aforementioned studies belongs to the G2b genogroup, we repeated the assays using another PEDV classical strain CV777 from the G1b genogroup to determine whether the mechanisms were strain-specific. The results demonstrated that palmitoylation of PEDV strain CV777 S protein also strengthened its stability through impeding recognition by HSC70 and antagonizing degradation via CMA to promote viral multiplication (Fig. S5). Collectively, these results demonstrate that palmitoylation of PEDV S protein promotes viral proliferation.

## DISCUSSION

In recent years, PED has become an epidemic around the world, causing extensive financial losses to the swine industry (6, 7). Therefore, it is urgent to comprehensively understand PEDV pathogenesis, which will provide novel insights into the prevention and control of PED. Palmitoylation is a significant PTM involved in multiple viral replication cycles (20–23, 40). Although palmitoylation of PEDV S protein has been reported to improve virion stability and membrane fusion for enhancing viral infection (41), its specific molecular mechanisms have not been fully elucidated. In the present study, we revealed that palmitoylation of PEDV S protein enhances its stability by impeding degradation via CMA to facilitate viral proliferation.

Palmitoylation involves the attachment of fatty acids to proteins (15). In our study, we verified that PEDV S protein was modified by S-palmitoylation through a biological orthogonal reaction and click chemistry (Fig. 1 and 7; Fig. S3, and S5). Previous studies on TGEV, MHV, SARS-CoV, and SARS-CoV-2 have also shown that their S proteins undergo palmitoylation (20–22, 24), suggesting that palmitoylation is a common PTM shared by CoV S proteins. Furthermore, we determined that the CRR within the PEDV S protein cytoplasmic tail was palmitoylated (Fig. 1). Palmitoylation is catalyzed by a family of enzymes known as ZDHHC palmitoyltransferases (42). We developed a siRNA library of ZDHHC enzymes and identified that ZDHHC5 mainly mediated palmitoylation of PEDV S protein (Fig. 2; Fig. S2). Interestingly, ZDHHC5 is also implicated in the palmitoylation of SARS-CoV-2 S protein (18, 20). We propose that ZDHHC5 may mediate the palmitoylation of various CoV S proteins, and consequently, ZDHHC5 can be developed into a promising target for anti-CoV therapeutics. In fact, we observed that ZDHHC1 and ZDHHC18 were involved in the palmitoylation of PEDV S protein as well (Fig. S2). Recent studies have also identified multiple palmitoyltransferases (ZDHHC5, ZDHHC9, and ZDHHC20) participating in the palmitoylation of SARS-CoV-2 S protein (18, 43). Therefore, we speculate that CoVs probably exploit redundant ZDHHCs for their own benefit.

Next, we substantiated that palmitoylation of PEDV S protein significantly strengthened its stability (Fig. 1 to 3). Of note, a recent study has mentioned that palmitoylation of SARS-CoV-2 S protein protects it from premature degradation to promote its biogenesis (43). However, the detailed molecular mechanisms have not been fully illustrated. In this study, we found that palmitoylation prevented the degradation of PEDV S protein via CMA (Fig. 4 to 6 and 8; Fig. S3 and S5). CMA is a selective protein degradation process in which target proteins containing KFERQ-like motifs are recognized by HSC70 and directed by LAMP2A into lysosomes (37, 38). In this study, we identified a functional KFERQ-like motif (QVDRL) in PEDV S protein and corroborated its interaction with HSC70, which mediated S protein degradation (Fig. 4 and 5). In contrast, mutation of the QVDRL motif impeded their interaction and prevented S protein from degradation (Fig. 4 and 5). Moreover, we proved that *HSC70* and *LAMP2A* knockdown effectively reversed the palmitoylation deficiency-induced degradation of PEDV S protein (Fig. 5 and 6). Taken together, we illuminated that palmitoylation of PEDV S protein prevents its recognition by HSC70 and restrains its degradation via CMA. In fact, we have analyzed the amino acid sequences of S proteins from four CoV genera. Importantly, we discovered that all CoV S proteins not only contain a CRR in their cytoplasmic tails but also possess one to three potential KFERQ-like motifs (Fig. S6; Table S1). Therefore, we speculate that the mechanism as described for PEDV is generic for all CoVs, which deserves further verification.

Finally, we proved that 2-BP significantly inhibited PEDV proliferation (Fig. 9; Fig. S4 and S5), and 2-BP treatment has been shown to inhibit other CoV propagation, such as TGEV, swine acute diarrhea syndrome coronavirus, MHV, and SARS-CoV-2 (21, 22, 40, 44). These data underline the significance of palmitoylation for CoVs. Despite different explanations being proposed, our study provided that palmitoylation deficiency led to the degradation of PEDV S protein through CMA, thereby inhibiting viral proliferation (Fig. 8 and 9; Fig. S3 to S5). We actually tried to construct a recombinant PEDV with deletion of the CRR in its S protein but failed to rescue it, indicating that the CRR is critical for PEDV viability. A previous study on MHV has also shown that deletion of the CRR is lethal for recombinant virus (45), which highlights once again that palmitoylation of S proteins is important for CoVs.

**Fig 10:**
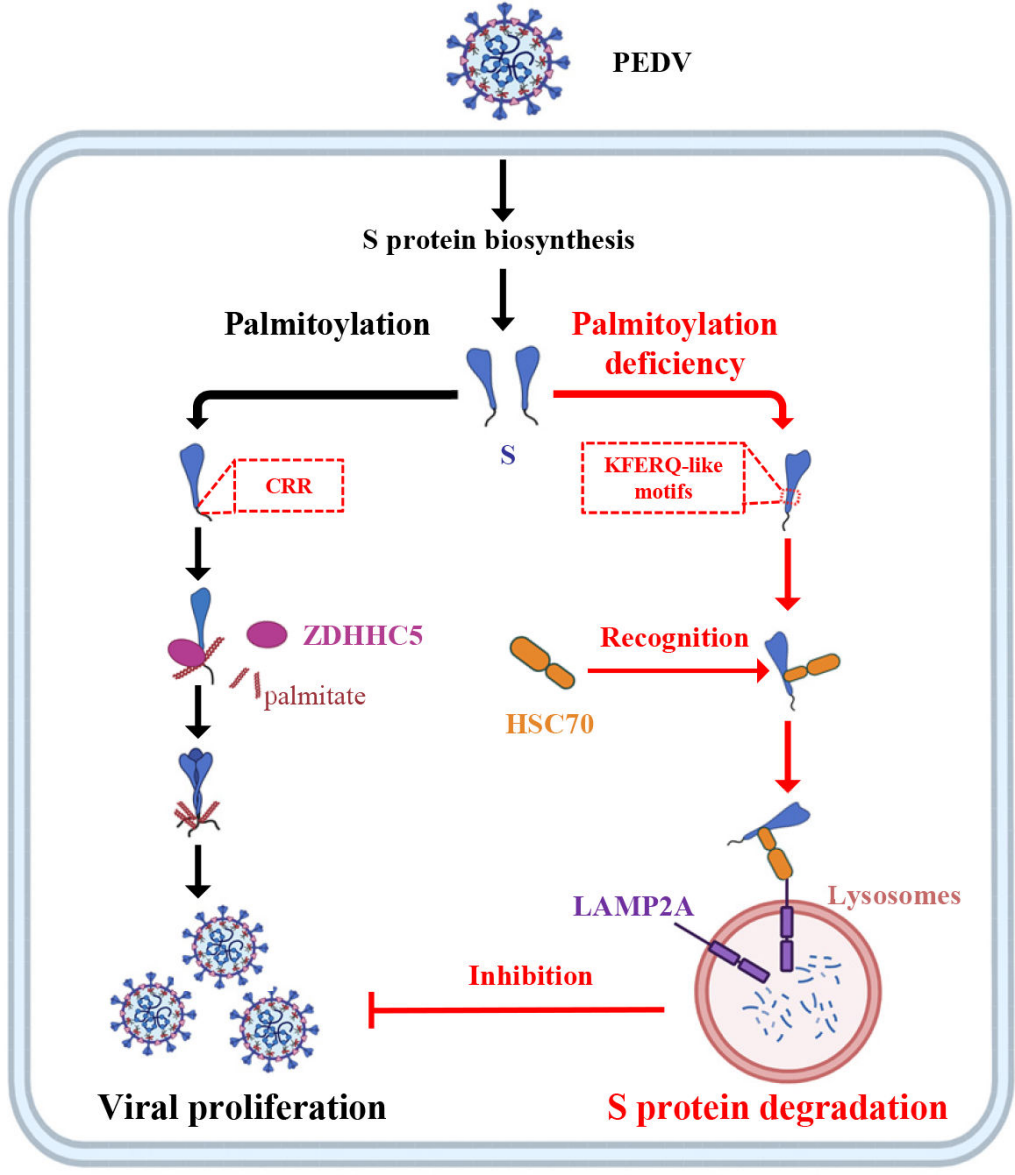
Schematic model depicting that palmitoylation of PEDV S protein strengthens its stability by antagonism of degradation via CMA, thus promoting viral proliferation. Mechanistically, palmitoylation of the CRR in PEDV S protein cytoplasmic tail is mediated by ZDHHC5 and prevents HSC70 from recognizing the KFERQ-like motif in S protein to antagonize its degradation through the lysosomal pathway, thereby strengthening its stability and facilitating PEDV proliferation.

In conclusion, we elucidate a molecular mechanism by which palmitoylation of PEDV S protein strengthens its stability by antagonism of degradation via CMA, thus promoting viral proliferation (Fig. 10). Our study actually deepens the understanding of PEDV pathogenesis and provides novel antiviral targets.

## MATERIALS AND METHODS

### Cells and viruses

Vero, IPEC-J2, and HEK-293T cells were stored in our laboratory (46, 47). Vero, IPEC-J2, and HEK-293T cells were maintained in Dulbecco’s modified Eagle’s medium (DMEM; Sigma-Aldrich, Cat. No. D5796) supplemented with 10% fetal bovine serum (FBS; Gibco, Cat. No. 10270–106) and penicillin-streptomycin liquid (Solarbio, Cat. No. P1400) at 37°C in a humidified incubator with a 5% CO_2_ atmosphere. PEDV strains CH/hubei/2016 (GenBank accession number: KY928065.1) and CV777 (GenBank accession number: KT323979.1) were utilized in the current study. PEDV strain CH/hubei/2016 belongs to the G2b genogroup (the non-S INDEL strain) and is prevalent in central China in recent years (48, 49). PEDV strain CV777 belongs to the G1b genogroup (the classical strain) (49, 50). The two strains were kept in our laboratory and were propagated in Vero or IPEC-J2 cells in FBS-free medium containing 6 µg/mL trypsin.

### Antibodies and reagents

Mouse anti-Flag monoclonal antibody (mAb; Cat. No. F1804) was purchased from Sigma-Aldrich. Mouse anti-Myc-tag mAb (Cat. No. 2276) was purchased from Cell Signaling Technology. Mouse anti-glyceraldehyde-3-phosphate dehydrogenase (GAPDH) mAb (Cat. No. 60004–1-Ig), rabbit anti-HSC70 polyclonal antibodies (pAbs; Cat. No. 10654–1-AP), rabbit anti-ZDHHC5 pAbs (Cat. No. 21324–1-AP), rabbit anti-ZDHHC5 Ab (Cat. No. 84803–4-RR), rabbit anti-LAMP2A pAbs (Cat. No. 27823–1-AP), and mouse anti-LAMP2 mAb (Cat. No. 66301–1-Ig) were purchased from Proteintech. Alexa Fluor 488-rabbit anti-calreticulin mAb (Cat. No. ab196158), DyLight 488-goat anti-mouse IgA antibody (Cat. No. ab97011), and Alexa Fluor 568-donkey anti-rabbit IgG (Cat. No. ab175470) were purchased from Abcam. Horseradish peroxidase (HRP)-labeled goat anti-rabbit IgG antibody (Cat. No. ab6721) and HRP-labeled goat anti-mouse IgG antibody (Cat. No. ab6789) were purchased from Jackson. Alexa Fluor 488-goat anti-mouse IgG antibody (Cat. No. A-11029), Alexa Fluor 647-goat anti-mouse IgG antibody (Cat. No. A-21235), and Alexa Fluor 488-goat anti-rabbit IgG antibody (Cat. No. A-11008) were purchased from Invitrogen. Mouse Isotype IgG (Cat. No. A7028) was purchased from Beyotime Biotechnology. Mouse anti-PEDV S protein mAb was kept in our laboratory (50). Mouse anti-PEDV N protein mAb (Cat. No. MPEDV-5A-2F7) was purchased from Immunology Consultants Laboratory.

Lipofectamine 2000 reagent (Cat. No. 2066194) and Lipofectamine RNAiMAX transfection reagent (Cat.No. 13778150) were purchased from Invitrogen. TransIntro PL transfection reagent (Cat. No. FT301-01) was purchased from TransGen Biotech. DMSO (Cat. No. D8371), trypsin (Cat. No. T8150), 4’,6-diamidino-2-phenylindole (DAPI; Cat. No. C0065), sucrose (Cat. No. S8271), phosphate-buffered solution (PBS; Solarbio, Cat. No. P1010), and paraformaldehyde (PFA; Cat. No. P1110) were purchased from Solarbio. Tris (2-carboxyethyl) phosphine hydrochloride (TCEP; Cat. No. ST049), phenylmethanesulfonyl fluoride (PMSF; Cat. No. ST505), Western blotting (WB)/IP lysis buffer (Cat. No. P0013), Triton X-100 (Cat. No. P0096), enhanced cell counting kit-8 (Cat.No. C0042), fast silver stain kit (Cat. No. P0017S), bovine serum albumin (BSA; Cat. No. ST025), anti-Flag magnetic beads (Cat. No. P2115), and anti-Myc magnetic beads (Cat. No. P2118) were purchased from Beyotime Biotechnology. Enhanced chemiluminescence (ECL) reagent (Cat. No. P0013B) and 0.25% trypsin-EDTA solution (Cat. No. C100C1) were purchased from NCM Biotechnology. Complete EDTA-free protease inhibitor cocktail (Cat. No. 04693116001) was purchased from Roche. PrimeScript RT master mix (Cat. No. RR036B), RNAiso Plus (Cat. No. 9109), and SDS loading buffer (Cat. No. 9173) were purchased from TaKaRa. ChamQ universal SYBR qPCR master mix (Cat. No. Q711) was purchased from Vazyme. CHX (Cat. No. HY-12320), 3-MA (Cat. No. HY-19312), carfilzomib (Cat. No. HY-10455), NH_4_Cl (Cat. No. HY-Y1269), CQ (Cat. No. HY-17589A), Alkynyl PA (Cat. No. HY-W040304), Tris(benzyltriazolylmethyl)amine (TBTA; Cat. No. HY-116677), biotin-azide (Cat. No. HY-129832), streptavidin magnetic beads (Cat. No. HY-K0208), and Protein G magnetic beads (Cat. No. HY-K0204) were purchased from MedchemExpress. Minute Lysosome isolation kit (Cat. No. LY-034) was purchased from Invent Biotechnologies. 2-BP (Cat. No. 21604), HAM (Cat. No. 467804), N-ethylmaleimide (NEM; Cat. No. 04259), and palmostatin B (Cat. No. 178501) were purchased from Sigma-Aldrich. Cupric sulfate anhydrous (CuSO_4_; Cat. No. C119000) was purchased from Aladdin. 7.5% SDS-PAGE (PG211) was purchased from Epizyme.

### Cell viability detection

Cell viability was detected by enhanced cell counting kit-8 according to the manufacturer’s instructions. Briefly, HEK-293T, Vero, and IPEC-J2 cells were seeded into 96-well plates and were incubated with 2-BP (0-100 µM), 3-MA (0.5–10 mM), carfilzomib (10–100 nM), NH_4_Cl (0.05–1 mM), CQ (10–100 μM), or DMSO for 48 h or transfected with si-*ZDHHC5* or si-NC for 48 h. The CCK-8 solution was added to each well, and the plates were incubated at 37°C for 1 h. The optical density at 450 nm was measured using a microplate reader (BioTek, Winooski, USA). The results are shown in Fig. S1.

### Inhibitor treatments

Vero cells were treated with or without 2-BP and incubated with 3-MA (2 mM), carfilzomib (20 nM), NH_4_Cl (0.5 mM), or CQ (40 µM), or DMSO for 36 h, and the cells were harvested for further analyses. Alternatively, Vero and IPEC-J2 cells were co-incubated with PEDV and non-cytotoxic 2-BP (2 µM, 4 µM, or 6 µM) or DMSO at 37°C for 24 h, and the cells were harvested for further analyses.

### RT-qPCR

Total RNAs were extracted using the RNAiso Plus, and cDNA was synthesized with the PrimeScript RT master mix kit. The cDNAs from different samples were amplified by PCR using ChamQ universal SYBR qPCR master mix on LightCycler480 II (Roche, Basel, Switzerland). The relative mRNA abundance of different samples was determined using the 2*^-^*^ΔΔCT^ method (51), with GAPDH mRNA as an endogenous control. The primers are listed in Table S2.

### IB

The protein samples were separated using 7.5% SDS-PAGE and transferred onto 0.2 µm polyvinylidene fluoride membranes (Merck Millipore, Cat. No. 03010040001). Next, the membranes were blocked by 5% skim milk in phosphate-buffered solution (PBS) with 0.05% Tween 20 (PBST) at room temperature (RT) for 2 h. The indicated primary antibodies were added and incubated with the membranes at 4°C overnight. After five washes with PBST, the membranes were probed with HRP-conjugated secondary antibodies and incubated at RT for 1 h. The immunoreactive bands were visualized using ECL reagent on a chemiluminescence imaging system (Fusion FX7; VILBER, Paris, France).

### RNA interference

All siRNAs and si-NC were designed and synthesized by GenePharma (Shanghai, China). The cells were transfected with the indicated siRNAs using Lipofectamine RNAiMAX according to the manufacturer’s instructions. The transfected cells were applied for subsequent experiments. The indicated siRNA sequences are listed in Table S3.

### Plasmid constructs and transfection

The genes of PEDV S protein (GenBank KY928065.1) were synthesized and cloned into the pCAGGS plasmid with Flag tag at the C-terminus by GENEWIZ (Suzhou, China). The cDNA of ZDHHC5 was amplified from Vero cells and constructed into the pCMV-N-Myc plasmid. The pcDNA3.1(+)/Myc-his A-HSC70 plasmid was kept in our laboratory (52). For the transfection, the plasmids were transfected with Lipofectamine 2000 or TransIntro PL transfection reagent according to the manufacturers’ recommendations.

### IP

Vero or IPEC-J2 cells were seeded into 60 mm dishes and infected with PEDV. Subsequently, the cells were washed with ice-cold PBS and lysed using WB/IP lysis buffer containing the protease inhibitor cocktail. The WCLs were centrifuged at 12,000 *g* for 10 min to collect the supernatant. To perform the IP assay, anti-PEDV S mAb or mouse isotype IgG was incubated with Protein G magnetic beads at RT for 1 h. Then, the beads were incubated with the supernatant at 4°C overnight. Finally, the samples were washed six times with PBST, eluted with SDS loading buffer, boiled at 100°C for 10 min, and measured by IB using the indicated antibodies.

### Co-IP

HEK-293T cells were transfected with the indicated plasmids in 60 mm dishes for 36 h. The cells were harvested and lysed by WB/IP lysis buffer supplemented with a protease inhibitor cocktail. The WCL was centrifuged at 12,000 *g* for 10 min to collect the supernatant, and 10% of the supernatant was separated as an input. The remaining lysates were incubated with 20 µL anti-Flag or anti-Myc magnetic beads at 4°C overnight. The beads were washed six times and eluted with SDS loading buffer at 100°C for 10 min. The samples were analyzed by IB using the indicated antibodies.

### S-Flag-IP/MS analysis

For the S-Flag-IP assay, Vero cells were transfected with the S-ΔCRR plasmid or Flag-tagged empty plasmid using TransIntro PL transfection reagent in 10 cm dishes for 36 h. The cells were washed with ice-cold PBS and lysed by WB/IP lysis buffer containing the protease inhibitor cocktail. The WCLs were centrifuged at 12,000 *g* for 10 min. Anti-Flag magnetic beads were incubated with the supernatant at 4°C overnight. The beads were washed six times with PBST. The associated proteins were analyzed by 7.5% SDS-PAGE, and the protein bands in the gel were stained with a fast silver stain kit. The indicated protein bands were cut and applied to LC-MS/MS by Lumingbio (Shanghai, China). The top-ranked peptide matches were taken into consideration for protein identification.

### IFA

Vero cells were washed with cold PBS and then fixed with −20°C methanol for 10 min. The cells were washed three times with cold PBS and blocked with 5% BSA in PBS at RT for 1 h. After being washed three times with cold PBS, the cells were probed with mouse anti-PEDV N mAb at RT for 2 h. After three washes with PBST, the cells were probed with the corresponding secondary antibodies and incubated at RT for 1 h. Cell nuclei were stained with DAPI for an additional 10 min. The fluorescent images were observed using a confocal laser scanning microscope (LSM800; Carl Zeiss AG, Oberkochen, Germany) with confocal laser scanning set up (10×). The ratio of PEDV-infected cells to total was calculated by ImageJ software (53, 54).

### Confocal microscopy

Vero cells were washed with cold PBS and then fixed with −20°C methanol for 10 min. The cells were washed three times with cold PBS and blocked with 5% BSA in PBS at RT for 1 h. After being washed three times with cold PBS, the cells were probed with the indicated primary antibodies at 4°C overnight. After three washes with PBST, the cells were probed with the corresponding secondary antibodies and incubated at RT for 1 h. Cell nuclei were stained with DAPI for an additional 10 min. Finally, the fluorescent images were observed using a confocal laser scanning microscope (LSM800; Carl Zeiss AG, Oberkochen, Germany) with confocal laser scanning set up (63×) and were representative as a single slice of a stack from three independent experiments (55). The co-localization analyses were performed using the JaCoP plugin in ImageJ software according to previous research guidelines (56–58). Pearson’s correlation coefficient (>0.5) and Manders’ overlap coefficient (>0.6) are considered to represent the true degree of co-localization and interaction. ImageJ software was used to measure and analyze the single-channel fluorescence intensity (56, 57).

### Metabolic labeling, click chemistry, and streptavidin pulldown

Metabolic labeling, click chemistry, and streptavidin pulldown were performed according to the published procedure with minor modifications (27, 59–61). Initially, cells were labeled for 8–12 h with either 50 µM Alkynyl PA in the DMEM supplemented with 10% FBS. Subsequently, the cells were collected by centrifugation, washed with cold PBS, and resuspended in lysis buffer (50 mM Tris-HCl pH 7.5, 150 mM NaCl, 1% Triton X-100, and 10% glycerol) supplemented with protease inhibitor cocktail, 1 µM palmostatin B, and 50 mM NEM. After lysis for 30 min, the lysate was centrifuged at 13,800 *g* at 4°C for 10 min. The collected supernatant was incubated with 50 µL anti-Flag magnetic beads or 10 µg anti-PEDV S mAb and 100 µL Protein G magnetic beads. After incubation, the beads were washed three times with lysis buffer and subjected to Cu(I)-assisted click reactions. The beads were incubated in PBS supplemented with 100 µM biotin-azide, 100 µM TBTA, 1 mM TCEP, and 1 mM CuSO_4_ for 1 h, protected from light, and washed five to six times with PBS. Next, the beads were separated, and the supernatant was discarded. 100 µL elution buffer (0.1 M glycine-HCl pH 2.7) was added to the beads, incubated at RT for 10 min, and added with 10 µL neutralization buffer (1 M Tris-HCl pH 9.0). The beads were then separated, and the supernatant was collected into a new EP tube. Streptavidin magnetic beads were incubated with the collected supernatant at 4°C overnight, followed by five to six washes with PBS with 0.05% Tween 20 (PBST). Finally, the samples were eluted with SDS loading buffer at 100°C for 10 min and analyzed by IB using the indicated antibodies.

### Lysosome isolation

Vero cells were transfected with the plasmid encoding PEDV S-Flag, S-ΔCRR, or Flag-tagged empty vector. At 6 h post-transfection, the S-Flag-overexpressed groups were treated with or without 20 µM 2-BP, and all the cells were treated with NH_4_Cl (0.5 mM) for 24 h. The cells were harvested and washed once with ice-cold PBS, followed by complete removal of the supernatant. Subsequently, lysosomes in these cells were isolated according to the instructions of the Minute Lysosome isolation kit. Finally, the samples were boiled with SDS loading buffer at 95°C for 10 min and subjected to IB with the indicated antibodies.

### CHX chase assay

Vero cells were transfected with the S-Flag plasmid and incubated with or without 20 µM 2-BP or transfected with the S-ΔCRR mutant. At 24 h post-transfection, the medium was discarded, and the cells were treated with 1 µg/mL CHX. The cells were collected and lysed at the indicated time points (0, 2, 4, 6, and 8 h). The samples were analyzed by IB.

### Virus purification

PEDV was propagated in Vero cells in FBS-free DMEM containing 6 µg/mL trypsin. After 24 h infection, culture supernatant was collected for virus purification (62). The prepared viral supernatant was centrifuged at 12,000 *g* at 4°C for 20 min to remove cell debris and then centrifuged at 150,000 *g* at 4°C for 120 min to concentrate viruses using a Beckman SW32Ti rotor. The viruses were suspended in PBS and purified using sucrose (10%–60%) gradient ultracentrifugation at 150,000 *g* for 120 min with the same rotor. Viral bands in sucrose solution were collected and centrifuged to remove sucrose. Finally, the purified viruses were resuspended in PBS buffer and stored at −80°C for later use.

### Virus titration assay

The infected cells were subjected to three freeze-thaw cycles, followed by centrifugation to remove cellular debris. The intracellular infectious virions were titrated by detecting TCID_50_ in Vero or IPEC-J2 cells. The infected cell supernatant was used to titrate extracellular infectious virions. Briefly, the Vero or IPEC-J2 cells seeded in 96-well plates were infected with a 10-fold serial dilution of PEDV at 37°C for 1 h. After removing the non-internalized viruses by washing with PBS, each well was added with 100 µL fresh medium and 6 µg/mL trypsin. Cytopathic effects caused by PEDV infection were observed using an inverted microscope (Axiovert 40, Carl Zeiss AG, Oberkochen, Germany) after 3 days. Finally, the TCID_50_ value was determined according to the Reed-Muench method (63).

### Statistical analysis

Three replicates were included in each experiment, and each experiment was independently repeated at least three times. The experimental data are presented as group means ± the standard errors of means (SEMs). All statistical analysis was carried out using Prism 8.0 software (San Diego, USA) with the unpaired two-tailed Student’s *t*-test. Statistical significance was indicated by asterisks (*P < 0.05; **P < 0.01; ***P < 0.001; ****P < 0.0001; ns, not significant [*P* > 0.05]).

## Data Availability

The data underlying this article will be shared on reasonable request to the corresponding authors.

## SUPPLEMENTAL MATERIAL

Supplemental material jvi.00347-25-s0001.docx Figures S1 to S6; Tables S1 to S3.

ASM does not own the copyrights to Supplemental Material that may be linked to, or accessed through, an article. The authors have granted ASM a non-exclusive, world-wide license to publish the Supplemental Material files. Please contact the corresponding author directly for reuse.
