# Supplementary material for: Palmitoylation enhances the stability of porcine epidemic diarrhea virus spike protein by antagonizing its degradation via chaperone-mediated autophagy to facilitate viral proliferation
Source: J Virol. 2025 May 22;99(6):e00347-25. doi: 10.1128/jvi.00347-25 (PMC12172468; doi:10.1128/jvi.00347-25)
Supplement: Supplemental material — Figures S1 to S6; Tables S1 to S3. [file jvi.00347-25-s0001.docx]

**SUPPLEMENTAL MATERIAL**

**FIGURES**


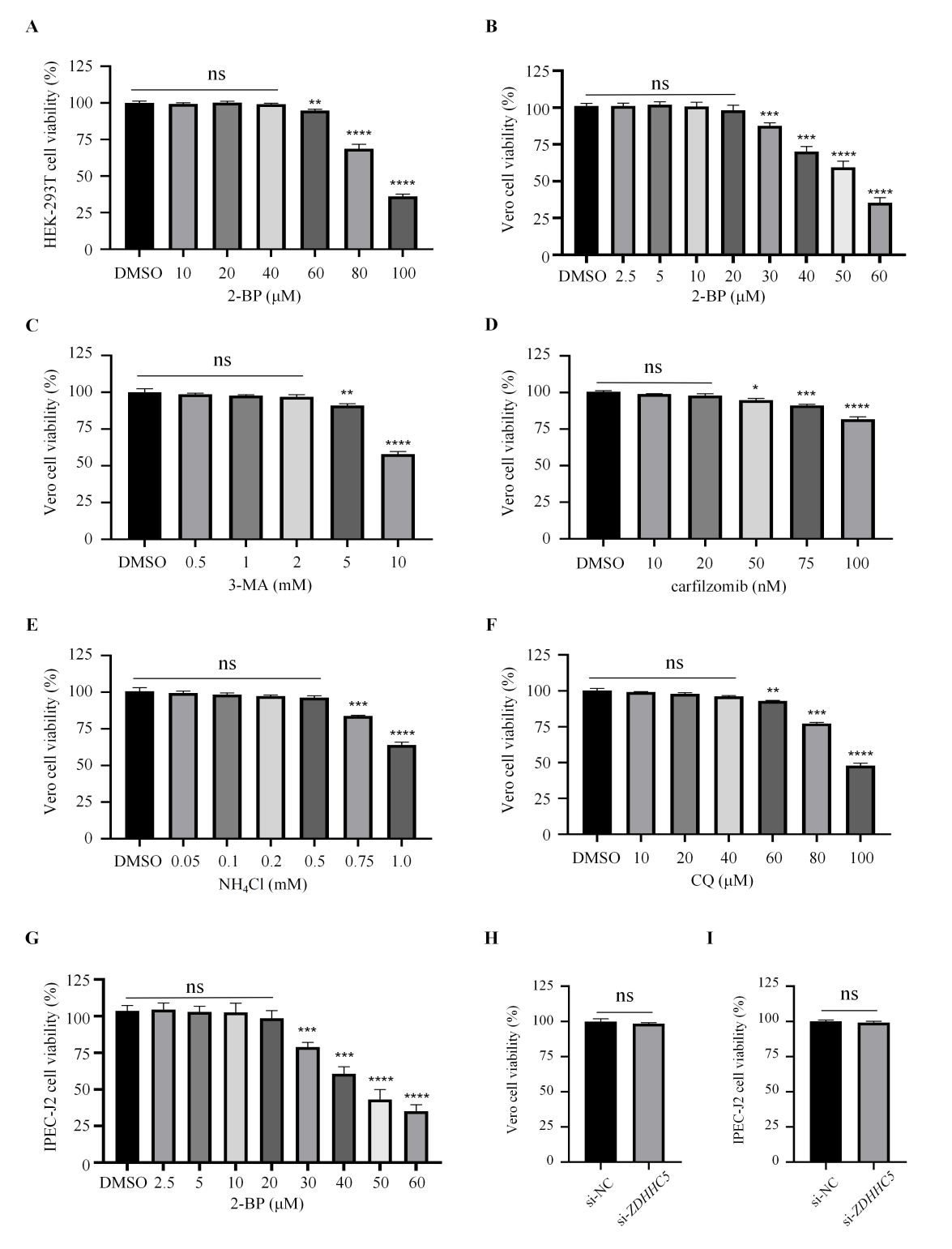


**FIG S1. Cell viability detection for the inhibitors and si-*ZDHHC5* in HEK-293T, Vero, and IPEC-J2 cells.** (**A**) HEK-293T cells were treated with 2-BP (10, 20, 40, 60, 80, or 100 μM) or DMSO for 48 h, and the cell viabilities were measured by enhanced cell counting kit-8. The cell viability in the DMSO-treated cells was set to 100%. (**B**-**F**) Vero cells were treated with 2-BP (2.5, 5, 10, 20, 30, 40, 50, or 60 μM), 3-MA (0.5, 1, 2, 5, and 10 mM), carfilzomib (10, 20, 50, 75, and 100 nM), NH_4_Cl (0.05, 0.1, 0.2, 0.5, 0.75, and 1 mM), CQ (10, 20, 40, 60, 80, and 100 μM), or DMSO for 48 h. Subsequent assays were performed as described in Fig. S1A. (**G**) IPEC-J2 cells were treated with 2-BP (2.5, 5, 10, 20, 30, 40, 50, or 60 μM) or DMSO for 48 h. Subsequent assays were performed as described in Fig. S1A. (**H** and **I**) Vero and IPEC-J2 cells were transfected with si-*ZDHHC5* or si-NC for 48 h, respectively. The cell viabilities were measured by enhanced cell counting kit-8. The cell viability in the si-NC-transfected cells was set to 100%. Statistical analysis was carried out using Student’s *t*-test. ns, not significant (*P* > 0.05); **P* < 0.05; ***P* < 0.01; ****P* < 0.001; *****P* < 0.0001.


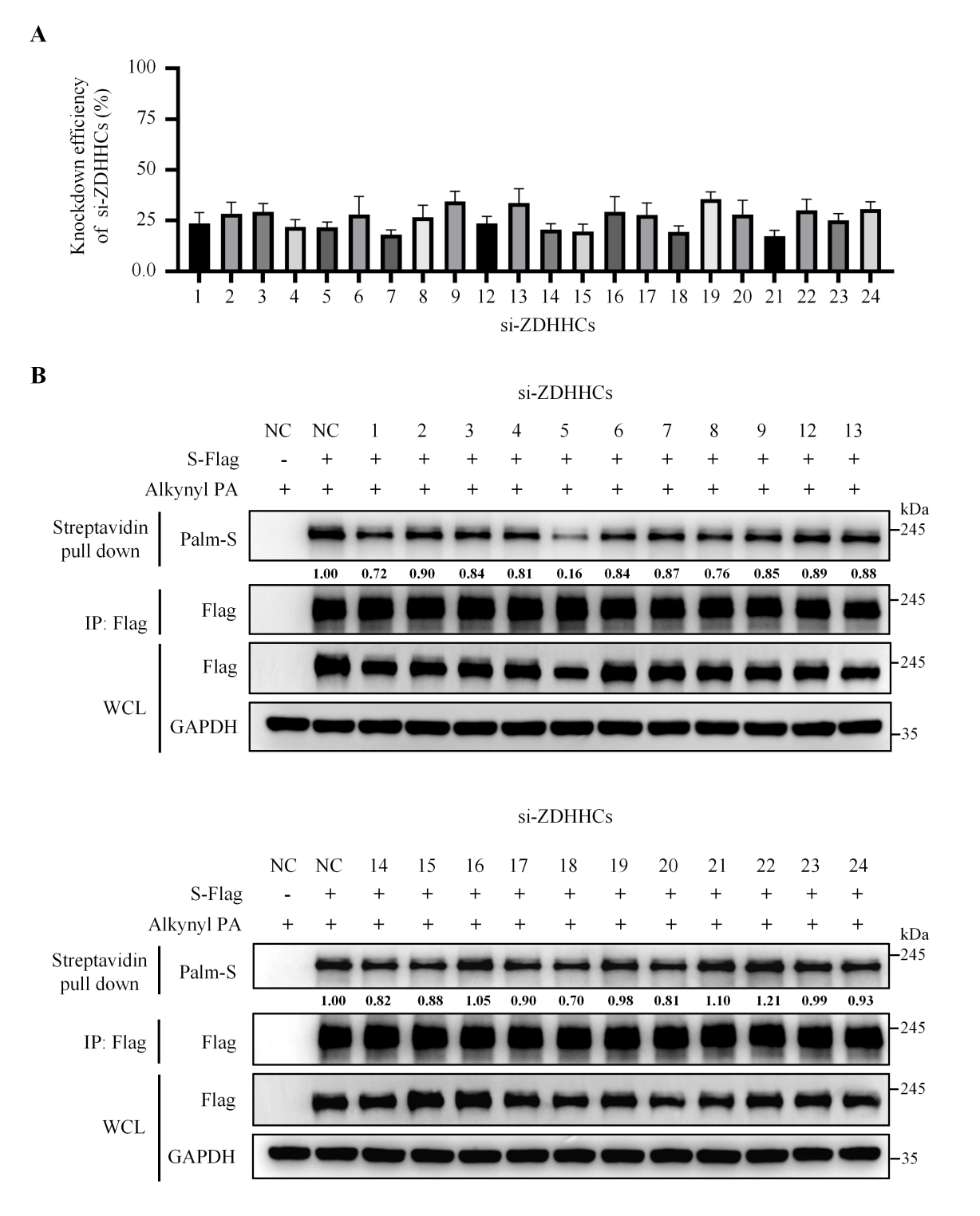


**FIG S2. ZDHHC5 mainly mediates palmitoylation of PEDV S protein.** (**A**) Vero cells were transfected si-*ZDHHC1-ZDHHC24* or si-NC for 36 h, and the knockdown efficiency of each si-ZDHHC was evaluated by RT-qPCR. Si-*ZDHHC11* was excluded as ZDHHC11 sequence is unavailable from the Genbank database. (**B**) Vero cells were transfected si-*ZDHHC1-ZDHHC24* or si-NC for 12 h, and were then transfected with the plasmid encoding PEDV S-Flag or Flag-tagged empty vector for 24 h. The cells were labeled with Alkynyl PA or DMSO as a control. The supernatant of WCL was immunoprecipitated using anti-Flag magnetic beads, followed by click chemistry and elution with glycine-HCl. The eluate was pulled down with streptavidin beads, and the precipitated proteins were analyzed by IB. The mean gray values of palmitoylated S protein were quantified using ImageJ software.


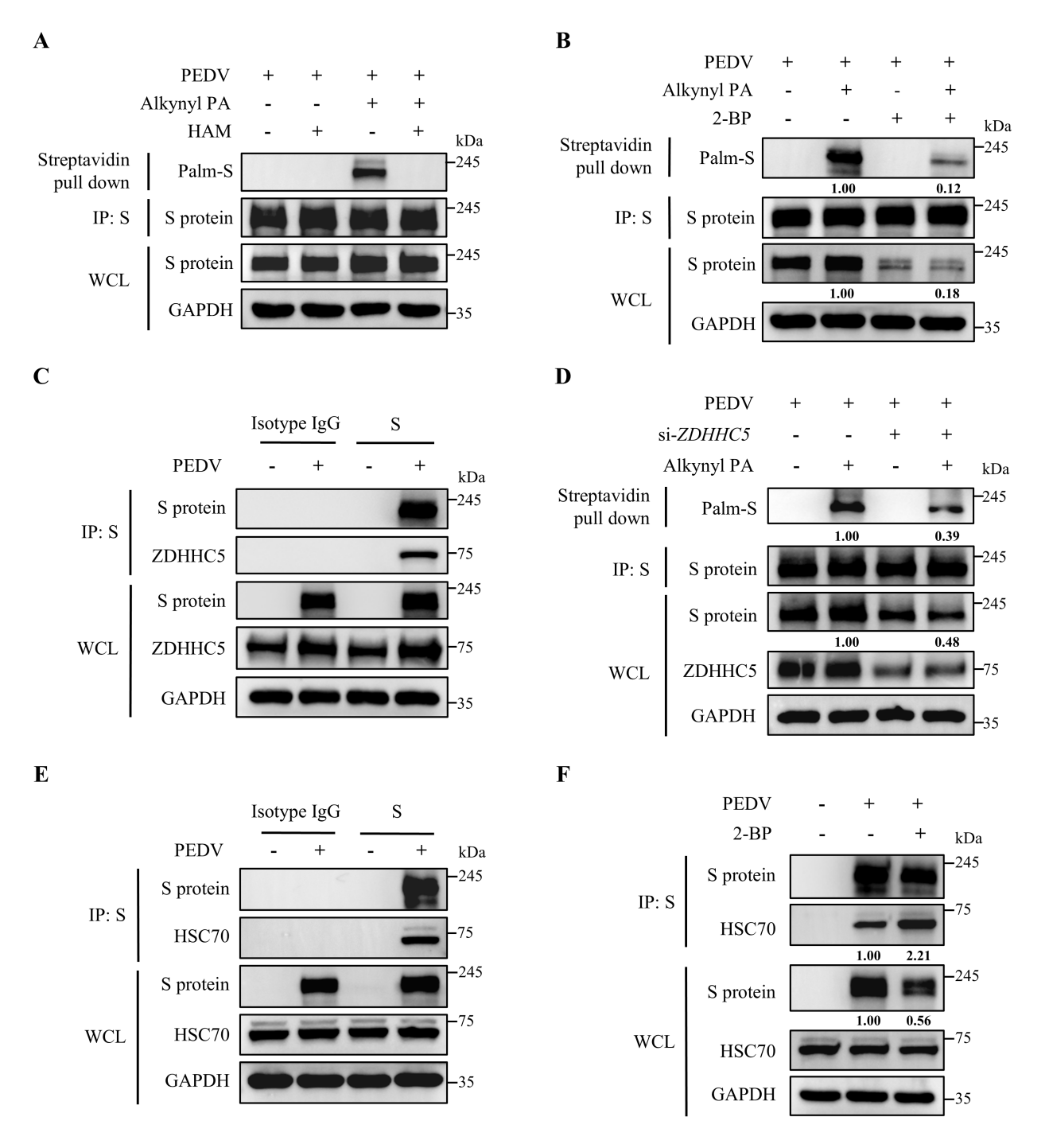


**FIG S3. Palmitoylation of PEDV S protein enhances its stability by antagonizing degradation via CMA in IPEC-J2 cells.** (**A**) IPEC-J2 cells were infected with PEDV at 0.1 MOI for 12 h and metabolically labeled with Alkynyl PA or DMSO as a control for 8 h. Subsequent assays were performed as described in Fig. 7A. (**B**) IPEC-J2 cells were infected with PEDV at 0.1 MOI for 12 h. The cells were treated with or without 6 μM 2-BP and metabolically labeled with Alkynyl PA or DMSO as a control for 8 h. Subsequent assays were performed as described in Fig. 7A. (**C**) IPEC-J2 cells were infected with PEDV at 0.5 MOI for 8 h and then lysed. Using S protein as bait, the precipitated proteins were analyzed by IB. Isotype IgG antibody was used as a negative control. (**D**) IPEC-J2 cells were transfected si-*ZDHHC5* or si-NC for 24 h, and were then infected with PEDV at 0.1 MOI for 12 h. The cells were treated with or without 6 μM 2-BP, and metabolically labeled with Alkynyl PA or DMSO as a control for 8 h. Subsequent assays were performed as described in Fig. 7A. (**E**) IPEC-J2 cells were infected with PEDV at 0.5 MOI for 8 h and then lysed. The supernatant of WCL was immunoprecipitated with anti-S mAb, and the precipitated samples were analyzed by IB. Isotype IgG antibody was used as a negative control. (**F**) IPEC-J2 cells were mock-infected or infected by PEDV at 0.5 MOI and treated with or without 6 μM 2-BP for 8 h. The supernatant of WCL was immunoprecipitated with anti-S mAb, and the eluted samples were measured by IB. The mean gray values of S protein and HSC70 were quantified using ImageJ software.


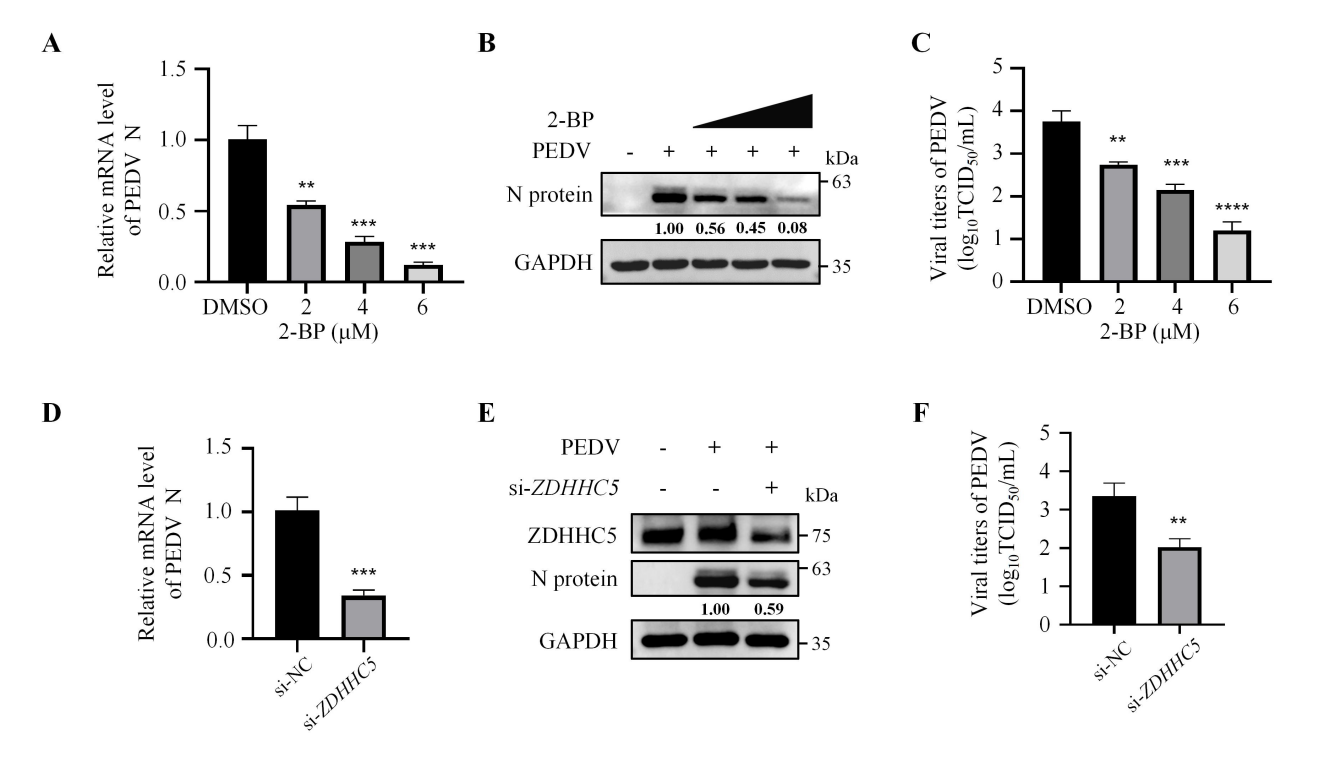


**FIG S4. Palmitoylation of PEDV S protein is critical for viral proliferation in IPEC-J2 cells.** (**A-C**) IPEC-J2 cells were mock-infected or infected by PEDV at 0.1 MOI and treated with 2-BP (2 μM, 4 μM, or 6 μM) or DMSO for 24 h. (**D-F**) IPEC-J2 cells were transfected with si-*ZDHHC5* or si-NC for 24 h, and incubated with PEDV for 24 h. (**A** and **D**) The viral RNA abundance was detected using RT-qPCR. (**B** and **E**) The viral N protein level was analyzed by IB. (**C** and **F**) PEDV titers were measured by assessing TCID_50_. The mean gray values of N protein were quantified using ImageJ software. Statistical analysis was carried out using Student’s *t*-test. ***P* < 0.01; ****P* < 0.001; *****P* < 0.0001.


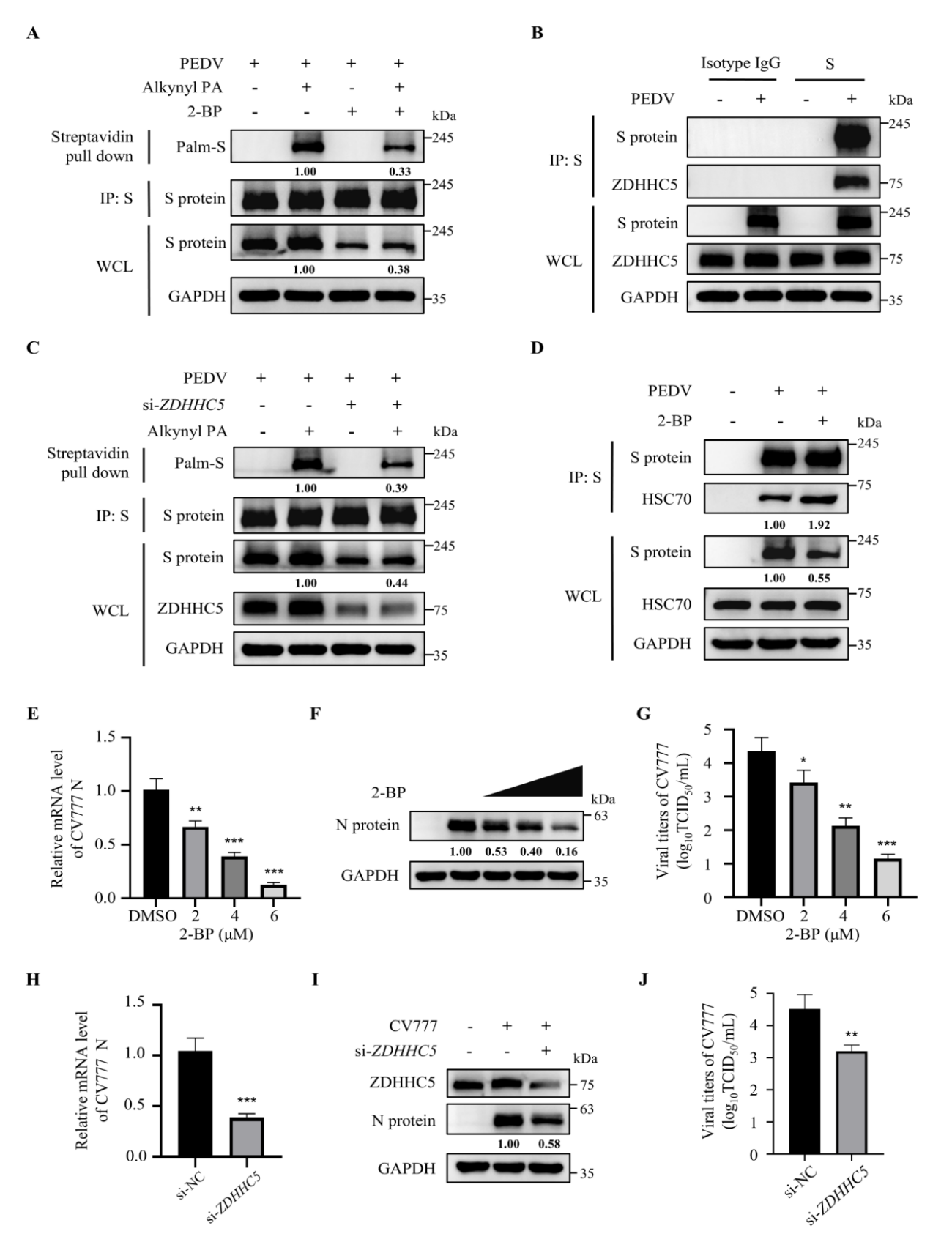


**FIG S5. Palmitoylation of PEDV strain CV777 S protein enhances its stability by antagonizing degradation via CMA to promote viral proliferation in IPEC-J2 cells.** (**A**) IPEC-J2 cells were infected with PEDV strain CV777 at 0.1 MOI for 12 h. The cells were treated with or without 6 μM 2-BP and metabolically labeled with Alkynyl PA or DMSO as a control for 8 h. Subsequent assays were performed as described in Fig. 7A. (**B**) IPEC-J2 cells were infected with PEDV strain CV777 at 0.5 MOI for 8 h and then lysed. Using S protein as bait, the precipitated proteins were analyzed by IB. Isotype IgG antibody was used as a negative control. (**C**) IPEC-J2 cells were transfected si-*ZDHHC5* or si-NC for 24 h, and were then infected with PEDV strain CV777 at 0.1 MOI for 12 h. The cells were treated with or without 6 μM 2-BP, and metabolically labeled with Alkynyl PA or DMSO as a control for 8 h. Subsequent assays were performed as described in Fig. 7A. (**D**) IPEC-J2 cells were mock-infected or infected by PEDV strain CV777 at 0.5 MOI and treated with or without 6 μM 2-BP for 8 h. The supernatant of WCL was immunoprecipitated with anti-S mAb, and the precipitated proteins were measured by IB. (**E-G**) IPEC-J2 cells were mock-infected or infected with PEDV strain CV777 at 0.1 MOI and incubated with 2-BP (2 μM, 4 μM, or 6 μM) or DMSO for 24 h. (**H-J**) IPEC-J2 cells were transfected with si-*ZDHHC5* or si-NC for 24 h, and incubated with PEDV strain CV777 at 0.1 MOI for 24 h. (**E** and **H**) The viral RNA abundance was detected using RT-qPCR. (**F** and **I**) The viral N protein level was analyzed by IB. (**G** and **J**) PEDV titers were measured by assessing TCID_50_. The mean gray values of S protein, HSC70, and N protein were quantified using ImageJ software. Statistical analysis was carried out using Student’s *t*-test. ns, not significant (*P* > 0.05); **P* < 0.05; ***P* < 0.01; ****P* < 0.001.


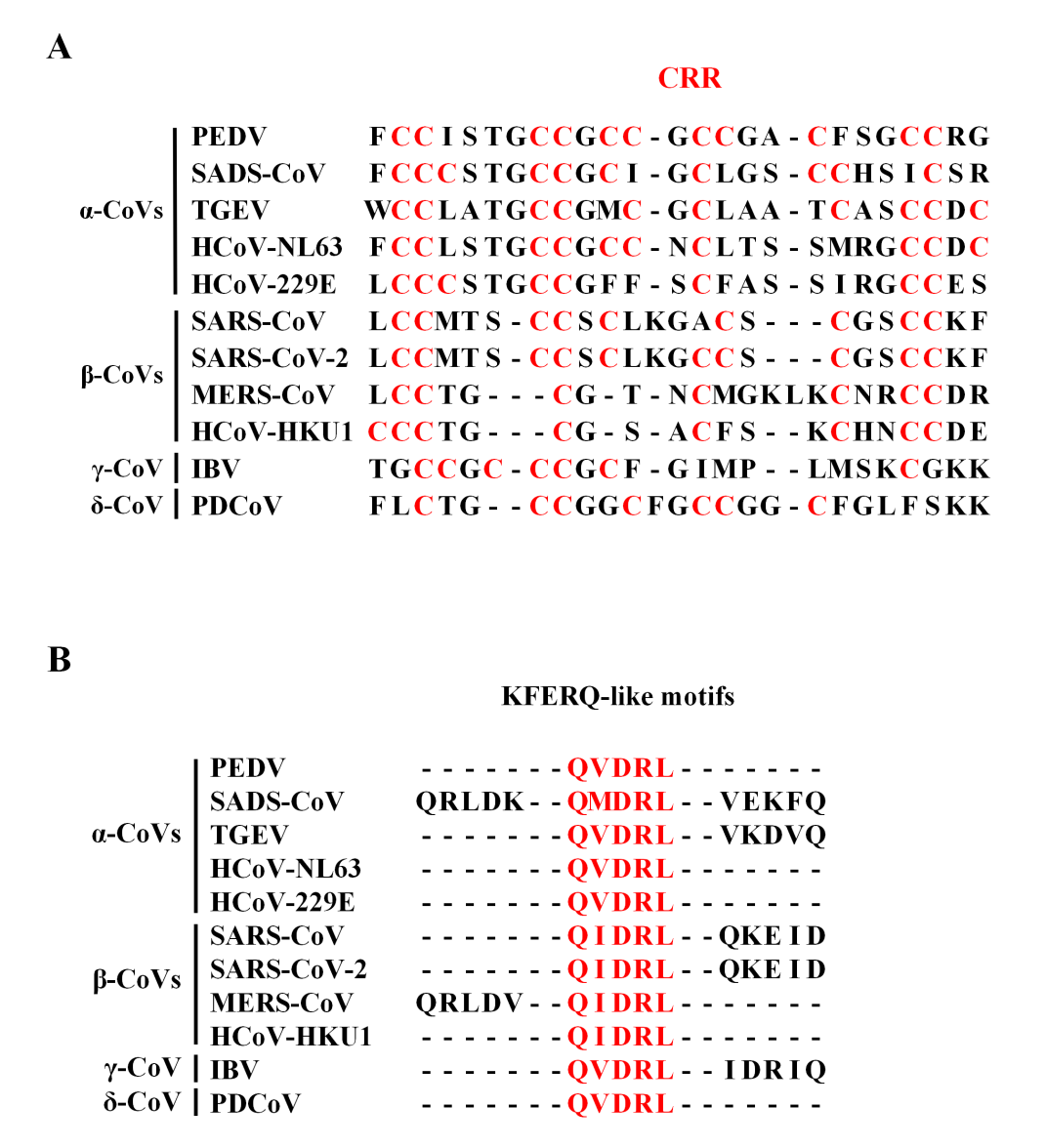


**FIG S6. Analyses of palmitoylation sites and KFERQ-like motifs in CoV S proteins.** Abbreviations: α-CoVs, alphacoronaviruses; β-CoVs, betacoronaviruses; γ-CoV, gammacoronavirus; δ-CoV, deltacoronavirus; SADS-CoV, swine acute diarrhoea syndrome coronavirus; HCoV-NL63, human coronavirus NL63; HCoV-229E, human coronavirus 229E; MERS-CoV, Middle East respiratory syndrome coronavirus; HCoV-HKU1, human coronavirus HKU1; IBV, infectious bronchitis virus; PDCoV, porcine deltacoronavirus.

TABLES

TABLE S1. The sequence information of strains involved in this study.

| Strain name | GenBank accession number | Strain name | GenBank accession number | Strain name | GenBank accession number |
| --- | --- | --- | --- | --- | --- |
| SM98 | GU937797.1 | LZC | EF185992.1 | attenuated DR13 | JQ023162.1 |
| CV777 | KT323979.1 | USA/Colorado/2013 | KF272920.1 | PC21A | KR078299.1 |
| CH/HLJGS/2023 | OR587951.1 | HN2021 | OK584017.1 | AJ1102 | JX188454.1 |
| JX2020 | MW386982.1 | CH/HLJBQL/2022 | OM914738.1 | CH/hubei/2016 | KY928065.1 |
| TGEV | NC_038861.1 | SADS-CoV | MG557844.1 | HCoV-229E | NC_002645.1 |
| HCoV-NL63 | NC_005831.2 | SARS-CoV | NC_004718.3 | SARS-CoV-2 | OQ299609.1 |
| MERS-CoV | KJ156934.1 | HCoV-HKU1 | NC_006577.2 | IBV | NC_001451.1 |
| PDCoV | MZ388470.1 |  |  |  |  |

TABLE S2. The primers for RT-qPCR used in this study.

| Target gene | Sequence (5’-3’) | |
| --- | --- | --- |
|  | Sense | Antisense |
| Monkey-GAPDH | TGACAACAGCCTCAAGATCG | GTCTTCTGGGTGGCAGTGAT |
| Pig-GAPDH | ATGTTTGTGATGGGCGTGAAC | CAGTCTTCTGGGTGGCAGTGAT |
| PEDV-N | CGCAAAGACTGAACCCACTAAC | TTGCCTCTGTTGTTACTTGGAGAT |
| PEDV-S | GCCTGACCTACTTCTGGCTGTT | CTGTTCACGCCTTGGTTCTC |
| Monkey-ZDHHC1 | CGAGTCATGTCCTCCCAAGAT | CACGGGTGGCAAGAAACTG |
| Monkey-ZDHHC2 | CTGGTCCTACTACGCCTACG | GGATTCATTGGTAATGTAAAGAT |
| Monkey-ZDHHC3 | ACTGTCCGTGGGTCAACAACT | AAAGCACAGCAGGATGAGGAG |
| Monkey-ZDHHC4 | TGTGTAACCAATCCTGGCATTAT | ACAGTGATGGTCGAAACGGT |
| Monkey-ZDHHC5 | GCCTGGCTACTAATGAGGATAG | GCTGATGTACTGCTGCTACTGT |
| Monkey-ZDHHC6 | AGCCACAAGAGGTTTACGATAC | TCAGCATCACAGGGACACTTT |
| Monkey-ZDHHC7 | GTCGTCCCACCTGAGAACCAT | CAGCACTTCGGGCACTTGTAG |
| Monkey-ZDHHC8 | GCACCCTCTTCTTCGTGTTCA | GTCCTCATCCTCATCCGCTCG |
| Monkey-ZDHHC9 | CACACTTTCCTCGTGGCTCT | CCTTCGATCCAGCACACTG |
| Monkey-ZDHHC12 | TGCTGACCTGGGGGATCA | CATGAGTGACACAGCGAGGTA |
| Monkey-ZDHHC13 | TGGACGGTGCATAGGTTTTG | AGGGGAACAGGCCACAATC |
| Monkey-ZDHHC14 | CCACGTCATTCTTCGTTCACA | GCCAACGATGGACCAGACAG |
| Monkey-ZDHHC15 | GTGCTCGTTATTGTCCTCGTC | TGCTGTGGGAGTGTAAAGATAGA |
| Monkey-ZDHHC16 | CAGGCCAAGGGCAGAGTATTTA | GAGGGGGCTCCCAGTTCAT |
| Monkey-ZDHHC17 | ACCTCTTGGACGGAAAACTC | ACCTGCTTCCACCAATTCTC |
| Monkey-ZDHHC18 | CGTCGCCTCCAACCTGACTA | ACGGTGTCGGACTGCACAA |
| Monkey-ZDHHC19 | CTGTTTGCTGCCTTCAATGTG | AGCGGAGCCTTGATGTAAGAT |
| Monkey-ZDHHC20 | TTCCCCCTCCAAAGAGTTCTAC | GTGATGTGCCCGATCAGGTT |
| Monkey-ZDHHC21 | ACACTCAACTAATTGGCATCATC | CCTCTGCCTGAAAGGAATGA |
| Monkey-ZDHHC22 | GCGGTCCTTTCCATCTCCTT | CACCTCCATCAGCCAGTTCT |
| Monkey-ZDHHC23 | GTTCTTACCTGCGGGTTATTTC | GCTGTGCGATTGTTGAGACTG |
| Monkey-ZDHHC24 | GCTCATCCTTCCCACCATTCT | CGAGGTCCTGCTGAGGTCAG |

TABLE S3. The siRNAs used in this study.

| Target gene | Sequence (5’-3’) | |
| --- | --- | --- |
|  | Sense | Antisense |
| Monkey-ZDHHC1 | GCACGCACAUGUCAUUGAATT | UUCAAUGACAUGUGCGUGCTT |
| Monkey-ZDHHC2 | CAAGCCAAGUUCCAUAUUATT | UAAUAUGGAACUUGGCUUGTT |
| Monkey-ZDHHC3 | GCGAGAACAACCAGAAGUATT | UACUUCUGGUUGUUCUCGCTT |
| Monkey-ZDHHC4 | GUGGUGAUGUCGGAUCUAUTT | AUAGAUCCGACAUCACCACTT |
| Monkey-ZDHHC5 | CCUGGGUGAACAACUGUAUTT | AUACAGUUGUUCACCCAGGTT |
| Monkey-ZDHHC6 | GAAGGUGUUUCAAGAAUAATT | UUAUUCUUGAAACACCUUCTT |
| Monkey-ZDHHC7 | CCGAAGUGCUGCUGUAUUATT | UAAUACAGCAGCACUUCGGTT |
| Monkey-ZDHHC8 | CCGUCUACAAUGGCAUCAUTT | AUGAUGCCAUUGUAGACGGTT |
| Monkey-ZDHHC9 | GGGACAUGUACACUCUUCUTT | AGAAGAGUGUACAUGUCCCTT |
| Monkey-ZDHHC12 | GGGAAUUCAUCUCCUCACATT | UGUGAGGAGAUGAAUUCCCTT |
| Monkey-ZDHHC13 | GGGAGAAACACCUCUUGAUTT | AUCAAGAGGUGUUUCUCCCTT |
| Monkey-ZDHHC14 | CCGAUCUGGAAAGGCAAAUTT | AUUUGCCUUUCCAGAUCGGTT |
| Monkey-ZDHHC15 | GGAGAUAAGAAGAAGUUCUTT | AGAACUUCUUCUUAUCUCCTT |
| Monkey-ZDHHC16 | GGUGCUCUUACCUUCUAGUTT | ACUAGAAGGUAAGAGCACCTT |
| Monkey-ZDHHC17 | GCUACACAAUAUGGAAUAUTT | AUAUUCCAUAUUGUGUAGCTT |
| Monkey-ZDHHC18 | CAGUGUCUGUGACAACUGUTT | ACAGUUGUCACAGACACUGTT |
| Monkey-ZDHHC19 | GACACCUUCACGGAUACAATT | UUGUAUCCGUGAAGGUGUCTT |
| Monkey-ZDHHC20 | GACGAAUGAACUGACAGAUTT | AUCUGUCAGUUCAUUCGUCTT |
| Monkey-ZDHHC21 | GGAAGAUCCUGUGGUUCAUTT | AUGAACCACAGGAUCUUCCTT |
| Monkey-ZDHHC22 | CAAGAGGUCUUCGGAAAGATT | UCUUUCCGAAGACCUCUUGTT |
| Monkey-ZDHHC23 | GCGGGUUAUUUCUGAUACUTT | AGUAUCAGAAAUAACCCGCTT |
| Monkey-ZDHHC24 | CCUUGCUGUUUGCUCGUAATT | UUACGAGCAAACAGCAAGGTT |
| Monkey-HSC70 | GGGACAAGGUAUCAUCAAATT | UUUGAUGAUACCUUGUCCCTT |
| Monkey-LAMP2A | GCUCUACUUAGACUCAAUATT | UAUUGAGUCUAAGUAGAGCTT |
| Pig-ZDHHC5 | UGAACAGGUUACGGGUAAATT | UUUACCCGUAACCUGUUCATT |
| si-NC | UUCUCCGAACGUGUCACGUTT | ACGUGACACGUUCGGAGAATT |
